# Limbic system synaptic dysfunctions associated with prion disease onset

**DOI:** 10.1186/s40478-024-01905-w

**Published:** 2024-12-20

**Authors:** Simote T. Foliaki, Bradley R. Groveman, Emmett A. Dews, Katie Williams, Hadil El Soufi, Benjamin Schwarz, Jacqueline M. Leung, Christine A. Schneider, Cindi L. Schwartz, Eric Bohrnsen, Cole D. Kimzey, Brent Race, Cathryn L. Haigh

**Affiliations:** 1 Laboratory of Neurological Infections and Immunity, National Institute of Allergy and Infectious Diseases, Division of Intramural Research, Rocky Mountain Laboratories, National Institutes of Health, Hamilton, MT USA; 2 Research Technologies Branch, National Institute of Allergy and Infectious Diseases, Division of Intramural Research, Rocky Mountain Laboratories, National Institutes of Health, Hamilton, MT USA

**Keywords:** Prion disease, Synaptic transmission, Neurodegeneration, Limbic system, Cognition, Emotion

## Abstract

Misfolding of normal prion protein (PrP^C^) to pathological isoforms (prions) causes prion diseases (PrDs) with clinical manifestations including cognitive decline and mood-related behavioral changes. Cognition and mood are linked to the neurophysiology of the limbic system. Little is known about how the disease affects the synaptic activity in brain parts associated with this system. We hypothesize that the dysfunction of synaptic transmission in the limbic regions correlates with the onset of reduced cognition and behavioral deficits. Here, we studied how prion infection in mice disrupts the synaptic function in three limbic regions, the hippocampus, hypothalamus, and amygdala, at a pre-clinical stage (mid-incubation period) and early clinical onset. PrD caused calcium flux dysregulation associated with lesser spontaneous synchronous neuronal firing and slowing neural oscillation at the pre-clinical stage in the hippocampal CA1, ventral medial hypothalamus, and basolateral amygdala (BLA). At clinical onset, synaptic transmission and synaptic plasticity became significantly disrupted. This correlated with a substantial depletion of the soluble prion protein, loss of total synapses, abnormal neurotransmitter levels and synaptic release, decline in synaptic vesicle recycling, and cytoskeletal damage. Further, the amygdala exhibited distinct disease-related changes in synaptic morphology and physiology compared with the other regions, but generally to a lesser degree, demonstrating how different rates of damage in the limbic system influence the evolution of clinical disease. Overall, PrD causes synaptic damage in three essential limbic regions starting at a preclinical stage and resulting in synaptic plasticity dysfunction correlated with early disease signs. Therapeutic drugs that alleviate these early neuronal dysfunctions may significantly delay clinical onset.

**Supplementary Information:**

The online version contains supplementary material available at 10.1186/s40478-024-01905-w.

## Introduction

Prion diseases (PrDs) are fatal neurodegenerative diseases caused by misfolding of normal prion protein (PrP^C^) into infectious isoforms, known as prions (PrP^Sc^). They affect humans, with Creutzfeldt Jakob disease (CJD) being the most common, and animals, including scrapie in sheep and goat, bovine spongiform encephalopathy (BSE) in cattle, and chronic wasting disease (CWD) in deer, elk, and moose. These disorders can arise spontaneously through unknown events (sporadic), or from inherited genetic mutation (familial) within the prion protein gene, or by infection (acquired). The infectious nature of prions has enabled the development of highly reproducible rodent models of acquired diseases, including mouse-adapted scrapie, which has been instrumental in our understanding of disease pathogenesis.

Clinical disease in mice inoculated with the RML strain of mouse-adapted scrapie begins at about ~ 70% of disease progression to terminal-stage disease (TSD), with reduced nesting and burrowing, followed by deteriorating motor deficit and weight loss [1–3]. Detailed behavioral assessments detect cognitive impairment, such as a lack of fear-induced memory formation early at clinical onset [1, 4]. Other abnormal signs, including increased glucose intake, urinary incontinence, and bladder enlargement, indicate endocrine system dysfunction [5]. Overt clinical signs that indicate terminal-stage disease include reduced mobility and severe ataxia, hunched posture, and loss of more than 15% of normal body weight [2, 5]. The mouse behavioral deficits have parallels in other species; for example, the symptoms reported in human diseases include psychotic features, agitated signs, and mood disorder [6]. Increased aggressive behavior has been reported in BSE and mink PrD [7].

The complex behavioral changes in PrD are linked to variable degrees of neural damage in various brain regions. In mice infected with RML prions, deep brain regions such as the hippocampus and thalamus have early prion deposits and more reactive glia than other regions [2, 8]. Further, the mechanism by which the disease damages synapses differs between the hippocampus and cerebellum [9]. Similarly, a distinct neuroinflammation pattern has been detected in the hippocampus, hypothalamus, cortex, and thalamus at an early stage of the disease [10]. In humans, proteomic analysis on brain tissues from sporadic CJD and various human genetic diseases has revealed more disease-associated protein changes in the cerebellum than in the cortex [11]. Consistently, brain magnetic resonance imaging (MRI) studies in CJD patients have shown some brain regions to have more abnormal hyperintense signals than others, including the cortex and thalamus [12].

Among the brain regions susceptible to PrD damage are the components of the limbic system, including the hippocampus, hypothalamus, and amygdala. These brain areas are essential for cognition and mood. PrD disrupts hippocampal plasticity in mice and this is associated with clinical onset [1, 13]. The neurotoxic scrapie RML prion has been shown to localize to the hypothalamic neurons and astrocytes [14], which is consistent with the neuroinflammation in this region [10] and with the heightened susceptibility of prion-infected hypothalamic GT1 neurons to oxidative stress [15]. Further, in some human genetic CJD cases (*PRNP* V180I mutation), the hippocampus and amygdala have been reported to have gliosis, vacuolation, and PrP deposition that is likely associated with synapses [16]. The disease-related damage of these limbic regions suggests that the limbic system is compromised during disease and this may be related to behavioral clinical signs.

Synaptic transmission is vital for neuronal network connectivity in the limbic system. Synapses are classified into excitatory such as the glutamatergic synapses, inhibitory including the GABAergic synapses, and modulatory comprising serotonergic and cholinergic synapses. Synaptic transmission can undergo a short-term change in response to stimuli by altering the neurotransmitter release and activity of neurotransmitter-gated ion channels, including NMDA receptors. The number and size of synapses can respond to stimulus with long-term changes, a phenomenon known as long-term synaptic plasticity, which is implicated in cognition [17]. This intrinsic plasticity of synapses is compromised in the hippocampus of prion-infected mice [13] and associated with prion neurotoxicity [18]. Long-term synaptic plasticity is also altered in the hippocampus of mice lacking PrP^C^ [19]. Together, this suggests a role of PrP^C^ in regulating synaptic transmission and plasticity [20].

Little is known about how prion disease affects synapses in the limbic regions and how that may contribute to the onset of early behavioral symptoms. We hypothesized that the dysfunction of synaptic transmission in the limbic system correlates with the onset of reduced cognition and behavioral abnormalities in scrapie-infected mice. We infected mice with RML prions and assessed neuronal physiology in three limbic regions, hippocampus, hypothalamus, and amygdala, at two disease time points, the mid-incubation period (a pre-clinical stage) and 70% of the disease progression to TSD, an early clinical onset stage. Our findings support that prion disease interferes with the plastic nature of synapses in the limbic system, compromising its higher-order functions correlating with the early onset of reduced cognition and behavioral deficits.

## Methods

### Animal ethics statement

All mice were housed at the Rocky Mountain Laboratories (RML) in an AAALAC-accredited facility in compliance with guidelines provided by the Guide for the Care and Use of Laboratory Animals (Institute for Laboratory Animal Research Council). All experiments were approved by the RML Animal Care and Use Committee under protocols 2019-043 and 2022–045.

### Prion inoculation

Six-week-old female wild-type C57BL10 mice were infected by injecting 30 µl of 1% (w/v) brain homogenate containing RML prion strains through the skull into parietal temporal lobes using a 27-gauge needle as described previously [21]. The inoculums were prepared from stocks previously determined to have a 50% infective dose (ID_50_) of 2.4 × 10^4^ RML. Uninfected control mice were ‘mock’ inoculated with 30 µl of 1% (w/v) uninfected brain homogenates. Mice were monitored twice weekly for observable clinical signs and every 1–3 days throughout the clinical phase. As described previously [2], the clinical phase starts with reduced nesting followed by hunchback posture, gait abnormalities, and ruffled coat appearance. The terminal disease stage was characterized by severe ataxia and weight loss, with an average incubation period of ~ 160 days post-inoculation (dpi). Mice were euthanized at ~ 50% (80 dpi) and 70% (108 dpi) of the disease progression to end-stage. At 80 dpi, mice showed no clinical sign, and at 108 dpi, all mice showed reduced nest-building behavior. All samples taken from these animals were handled and chemically decontaminated according to established scrapie protocols in consultation with RML Biosafety.

### Brain slice preparation

As described previously [18], 300 µm thick coronal brain sections containing the limbic regions of interest (hippocampus, hypothalamus, and amygdala) were collected from mice brains in an ice-cold cutting solution (3 mM KCl, 25 mM NaHCO_3_, 1.25 mM NaH_2_PO_4_, 206 mM Sucrose, 10.6 mM Glucose, 6 mM MgCl_2_·6H_2_O, 0.5 mM CaCl_2_·2H_2_O) using a vibratome (Leica VT1200S). The brain slices were incubated for 1 h at 32 °C in carbogenated (5% CO_2_; 95% O2) artificial cerebrospinal fluid (aCSF: 126 mM NaCl, 2.5 mM KCl, 26 mM NaHCO_3_, 1.25 mM NaH_2_PO_4_, 10 mM Glucose, 1.3 mM MgCl_2_·6H_2_O and 2.4 mM CaCl_2_·2H_2_O). The limbic regions were analyzed irrespective of their subregions, except for the electrophysiology recording, fluorescence microscopy, and transmission electron microscopy, which were specifically performed on hippocampal CA1, ventral medial hypothalamus (VMH), and basolateral amygdala (BLA).

### Electrophysiology recording

The electrophysiology recording was performed specifically on hippocampal CA1, VMH, and BLA. The slices were mounted on to 60MEA200/30iR-Ti-pr-T multi-electrode arrays (MEA; Multichannel Systems; Germany) for the electrophysiological recording while being continuously superfused with carbogenated aCSF, containing MgCl_2_ for recording with electric stimulation and without for recording of spontaneous synchronous neuronal firing.

For spontaneous recording of neuronal firing, local field potentials were recorded and filtered as described previously [22]. Population spikes were detected as those above four standard deviations of the mean noise using the Multichannel Systems Rack software (Multichannel Systems). A neuronal burst was characterized as at least 4 spikes within 100 ms. Neuronal bursts and raster plots were analyzed and generated by MEAnalyzer [23]. Oscillatory powers and peak frequency of delta (0.5–4 Hz) and gamma (33–80 Hz) oscillations were analyzed by MATLAB (R2021a) as described previously [24].

For recording of evoked synaptic transmission and plasticity, one of the electrodes located along each region of interest was used to stimulate neurons by applying electric stimulation (2000–2500 mV) to evoke the field excitatory postsynaptic potential (fEPSP). The baseline stimulation intensity was determined by an input–output curve obtained from stimulating populations of interconnected neurons with increasing strength of electric stimulation (input), starting at 500 mV to a stimulation intensity (4000–5000 mV) that evoked the maximum fEPSP amplitude (output). This max response was indicated by the point where the input–output curve reached a plateau. The baseline stimulation intensity was the baseline stimulation strength that evoked 30–50% of the maximum fEPSP amplitude. The baseline fEPSP was recorded for 5 min by applying the baseline stimulus in 30-s intervals. Immediately after the baseline recording, the long-term synaptic plasticity was induced by tetani, consisting of three 100 Hz trains (500 ms long per train) delivered in 20-s intervals. The post-tetani fEPSPs were recorded for 30 min, with the last ten-minute recording averaged and used for the statistical analysis of long-lasting plasticity. In a slice, all synaptic plasticity readings from electrodes with fEPSPs were averaged to represent that slice. To block NMDA receptors, slices were continuously superfused with 200 µM D-AP5 (Cayman Chem; Item No. 14539) in aCSF for the duration of the recording. The brain slices were snap-frozen and stored at − 80 °C or fixed in 10% formalin for further analyses. We utilized paired-pulse (PP) test to estimate presynaptic release (detailed description in [18]), whereby two similar strength stimuli were delivered 20 ms apart to generate two fEPSPs. The ratio (PPR) of these two pulses estimates the probability of release (*pr*) from the readily releasable pool of vesicles in an inverse-proportional manner. The higher the PPR, the lower the *pr*, and vice versa.

### Immunofluorescence

Mice brains were fixed in 10% (v/v) formalin, paraffin embedded, and sectioned into 5 μm-thick coronal slices containing hippocampus, amygdala and hypothalamus. The coronal sections were then deparaffinized, permeabilized with 0.1% Triton-X-100, blocked with 5% w/v bovine serum albumin (BSA), immunolabelled with primary antibodies or probes (Table 1) and appropriate secondary antibodies, mounted using ProLong Gold antifade agent (ThermoFisher Scientific), and imaged by an EVOS FL Auto Imaging System (Invitrogen) or confocal microscopy using a Zeiss laser scanning LSM 880 confocal microscope driven by ZEN v.2.3 software. The immunofluorescence images were quantified for statistical analysis using Image J 1.52n as described previously [22].

**Table 1:** Antibodies and probes

Antibodies	From	Catalog #	Use (Dilutions)	Host species	Secondary antibodies for WB (Dilutions)	Secondary antibodies for IF (Dilutions)
6D11	BioLegend	808,001	WB (1:5 K)	Mouse (Ms)	Goat (Gt) anti Ms HRP (1:5 K)	–
MAP2	SynapticSystems	188,006	IF(1:500)	Chicken (Ch)	–	Gt anti Ch Alexa Fluor 647 (1:500)
Synaptophysin	ABCAM	ab8049	WB (1:5 K)/IF(1:200)	Mouse	Gt anti Ms HRP (1:5 K)	Gt anti Ms Alexa Fluor 488 (1:500)
PSD95	ABCAM	ab18258	WB(1:5 K)/IF(1:200)	Rabbit (Rb)	Gt anti Rb HRP (1:5 K)	Gt anti Rb Alexa Fluor 488 (1:500)
Phalloidin	Invitrogen	A12379	IF(1:500)	–	–	–
NF-L	Invitrogen	13–0400	WB(1:5 K)/IF(1:200)	Mouse	Gt anti Ms HRP (1:5 K)	Gt anti Ms Alexa Fluor 568 (1:500)
Beta tubulin	ABCAM	ab6046	IF(1:200)	Rabbit	–	Gt anti Ms Alexa Fluor 647 (1:500)
Synaptojanin 1	ABCAM	ab308136	IF(1:100)/WB(1:1 K)	Rabbit	Gt anti Rb HRP (1:5 K)	Gt anti Rb Alexa Fluor 488 (1:500)
NeuN	ABCAM	ab177487	WB(1:2 K)	Rabbit	Gt anti Rb HRP (1:5 K)	–

### Confocal microscopy

Specific brain regions, including the CA1, VMH, and BLA were imaged using a Zeiss laser scanning confocal (LSM 880) microscope driven by ZEN v2.3 software (Carl Zeiss Microscopy). A Plan Apochromat 63X/NA1.4 oil immersion lens was used, with immersion oil at a refractive index of 1.518. Image acquisition settings including laser power and gain were optimized for minimal background and cross-talk, and kept constant within an experiment for all samples to enable direct comparisons. Stacks were collected with a lateral resolution of 37 nm and z-spacing of 373 nm to identify slices with optimal signals for further analysis. Huygens v. 22.10 (Huygens Professional) was used for image deconvolution and quantification of synaptic and cytoskeletal markers. In this quantification, the intensity of the marker of interest was measured in various fields of view that were selected randomly. Huygens was also used to perform segmentation on images labelled with MAP2 (dendritic spine), PSD95 (post synaptic density), and synaptophysin (pre synapse) to estimate synapse size, density, and formation by measuring the colocalization (by Pearson’s correlation) between pre- and post-synaptic density as well as post-synaptic density and dendric spines. The size of pre-synapse was also estimated by measuring particle area using ImageJ 1.52n [25] after binarizing the synaptophysin fluorescence signal.

### Calcium influx

Intracellular calcium level was measured by Fluo-4 Direct Calcium Kit (ThermoFisher; F10471) as described previously [22]. The limbic regions of interest were dissected out from fresh coronal brain sections and incubated in 50% (v/v, in aCSF) 2 × Fluo-4 Direct calcium reagent at 37 °C without CO_2_ for 40 min (in ClarioStar plate reader, BMG), while the fluorescence of the reagent was read at 494 nm excitation and 516 nm emission in a circular pattern every minute.

### RT-QuIC

RT-QuIC analysis was performed as previously described [26] with minor modifications. Briefly, indicated brain regions were isolated from brain slices, as prepared above, from pre-clinical or clinical mice. Individual brain regions were homogenized to 1% w/v in 1 × PBS and pre-cleared with a 2000×*g* centrifugation for 2 min. Supernatants were serially diluted by 10 folds in 0.1% sodium dodecyl sulfate (SDS)/PBS/N2. Dilutions from each sample were tested in quadruplicate reaction wells of a black clear bottom 384-well plate. Each well was loaded with 1 μL of sample dilution into 49 μL of reaction mix containing a final concentration of 10 mM phosphate buffer [pH 7.4], 300 mM NaCl, 0.1 mg/mL hamster recombinant PrP 90–231, 10 μM thioflavin T, and 1 mM ethylenediaminetetraacetic acid tetrasodium salt and 0.002% SDS (contributed by the sample dilution). Plates were sealed and incubated in a FLUOstar Omega plate reader (BMG) at 50 °C for 30 h with cycles of 60 s of shaking (700 rpm, double orbital) and 60 s of rest. ThT fluorescence was measured every 45 min (excitation, 450 ± 10 nm; emission, 480 ± 10 nm [bottom read]). Reaction wells were considered positive when they exceeded a threshold of 10% of the maximum value on each plate within the 30-h time cutoff. LogSD_50_/mg tissue was calculated using Spearman-Kärber analyses [27] to provide estimates of the concentrations of seeding activity units giving positive reactions in 50% of replicate reactions, i.e., the 50% “seeding doses” or SD50’s as previously described [26].

### Neurotransmitter measurement

Neurotransmitter level changes were assayed as previously described [24]. Liquid chromatography mass spectrometry (LCMS)-grade solvents were used throughout. Briefly, brain sections (n = 5 per section) were immediately submerged in 0.5 mL of ice-cold methanol and homogenized via bead milling. To the resultant homogenate was added a 0.5 mL of water and 0.5 mL of chloroform. Samples were agitated for 30 min under refrigeration and centrifuged at 16 k×*g* for 20 min. 400 µL each of the top (aqueous) layer was collected and diluted as necessary in 1:1 methanol:water for LCMS injection.

Neurotransmitters were collected as part of a broader targeted metabolomics dataset from a combination of two analytical methods with opposing ionization polarities in order to optimally target different classes of metabolites. For instance, more cation-prone metabolites such as those containing primary amines were assayed preferentially in positive mode while phosphorylated metabolites were preferentially assayed in negative mode. A series of metabolites with mixed ionization preference was assayed in both polarities and enabled both scaling, and confirmation of the quality of scaling, of the two resultant datasets such that they could be combined and analyzed together. Both methodologies utilized a LD40 XR UHPLC (Shimadzu Co.) system for separation and a 6500 + QTrap mass spectrometer (AB Sciex Pte. Ltd.) for detection. Negative mode samples were separated on a Waters™ Atlantis T3 column (100 Å, 3 µm, 3 mm × 100 mm) and a binary gradient from 5 mM tributylamine, 5 mM acetic acid in 2% isopropanol, 5% methanol, 93% water (v/v) to 100% isopropanol over 5 min. Positive polarity analysis utilized a Phenomenex Kinetex F5 column (100 Å, 2.6 µm, 100 × 2.1 mm) and a gradient from 0.1% formic acid in water to 0.1% formic acid in acetonitrile over 5 min. Both resultant datasets were integrated using SciexOS 3.1.6.44. Signal quality for each set of brain regions was assessed visually and signals with a signal to noise less than 5 were excluded for that brain region. Similarly, signals present in less than 50% of samples for a brain region were excluded. Positive and negative datasets were scaled together based on common signals for glutamine and this scaling was confirmed using a series of other amino acids with robust signals in both datasets. Datasets were normalized by the total sum of metabolite signals for each sample.

### Sarkosyl insolubility assay

As described previously [22], 20 µL of ~ 1% (w/v) brain homogenate was solubilized in 300 µL of 10% (w/v) Sodium lauroyl sarcosinate (sarkosyl) at room temperature with constant agitation at 1400 rpm for 30 min, diluted with 2680 µL of ice-cold homogenization buffer (10 mM Tris–HCl, 1 mM EGTA, 0.8 M NaCl, 10% sucrose, pH 7.4), and centrifuged at 100 K × g at 4 °C for 30 min. Supernatants and pellets were separated to isolate the sarkosyl soluble and insoluble proteins respectively. Soluble proteins in supernatants were precipitated by methanol precipitation. Four parts ice cold pure methanol and one part supernatant were mixed, incubated at -20 °C overnight, and centrifuged at 5000×*g* for 1 h at 4 °C to pellet all soluble proteins. The insoluble pellet and soluble pellets were resuspended in 2 × sample buffer containing ~ 1% (v/v) beta-mercaptoethanol (BME) for western blotting analysis of PrP.

### Western blotting

The three limbic regions were dissected out of fresh coronal brain sections and homogenized in 1 × PBS. The total protein concentration was corrected between samples by BCA assay. Protein samples containing ~ 1 × sample buffer and ~ 1.5% (v/v) BME were boiled at 100 °C for 5 min and loaded into Bolt 4–12% Bis–Tris Plus gels (Invitrogen). The proteins were resolved into various sizes ranging between 3 and 260 kDa as indicated by the Novex Sharp Pre-stained protein standard or SeeBlue Pre-stained protein standard (Life Technologies) and then transferred to a PVDF membrane (Millipore). The membrane was blocked with the EveryBlot Blocking Buffer (BIO-RAD) for 10 min at room temperature. The proteins of interest were labeled with primary antibodies and appropriate HRP conjugate secondary antibodies (see supplementary file 1), which were detected by ECL Select (Amersham) and imaged by iBright imaging system (Invitrogen). The densitometry analysis was performed using Image J 1.52n, and the band density was normalized to the total protein density (by Coomassie stain) as the loading control.

### Transmission electron microscopy

Sections of mouse brain were fixed in 4% paraformaldehyde, 2.5% glutaraldehyde in 0.1 M Sorenson’s phosphate buffer (SPB) (Karnovsky’s Fixative, Electron Microscopy Sciences) and stored at 4 °C until further processed. Sections were rinsed three times in 0.1 M SPB, post-fixed for one hour in 0.5% OsO4, 0.8% K4Fe(CN)6 in 0.1 M SPB, rinsed three times in dH2O, stained with 1% aqueous tannic acid for one hour, rinsed three times with dH2O, and then stained en bloc in 1% aqueous uranyl acetate for one hour. Samples were rinsed three times with dH2O followed by dehydration by a graduated ethanol series into 100% ethanol. Sections were embedded in eponate resin over several days into 100% resin. Blocks were polymerized at 60 °C for 3 days. 70 nm sections were cut and placed onto Formvar coated 100 hexagonal mesh copper grids (Electron Microscopy Sciences) for imaging in a Hitachi 7800 TEM operating at 80 kV. Images or fields of view were acquired on an AMT XR81-B digital camera. Synapses were identified in each high-magnification image and classified as normal or abnormal, as indicated by damaged post-synapses and depleted and deformed pre-synaptic vesicles. Damaged synapses were identified by disruption of synaptic membrane, collapse of pre-synapse to post-synapse, excessive accumulation of proteins in synaptic cleft, and thinning of synaptic membrane lining. Synapses with depleted synaptic vesicles were identified by (1) loss of vesicles at the readily releasable pool (the pool of vesicles at the release site) relative to the reserve pool as well as (2) decline in vesicles with clear morphology at the release site. The counts of images with abnormal synapses were normalized to the total number of images.

### Data analysis

Unless otherwise stated, data are presented showing independent ‘n’ (mouse brain) values as individual dots with bars indicating mean and error bars showing the standard error of mean. All statistical analyses were conducted using GraphPad Prism 8, and statistical methods used are listed in figure legends.

## Results

### Prion propagation in the limbic regions is associated with the transition from pre-clinical to clinical stage

**Fig. 1:**
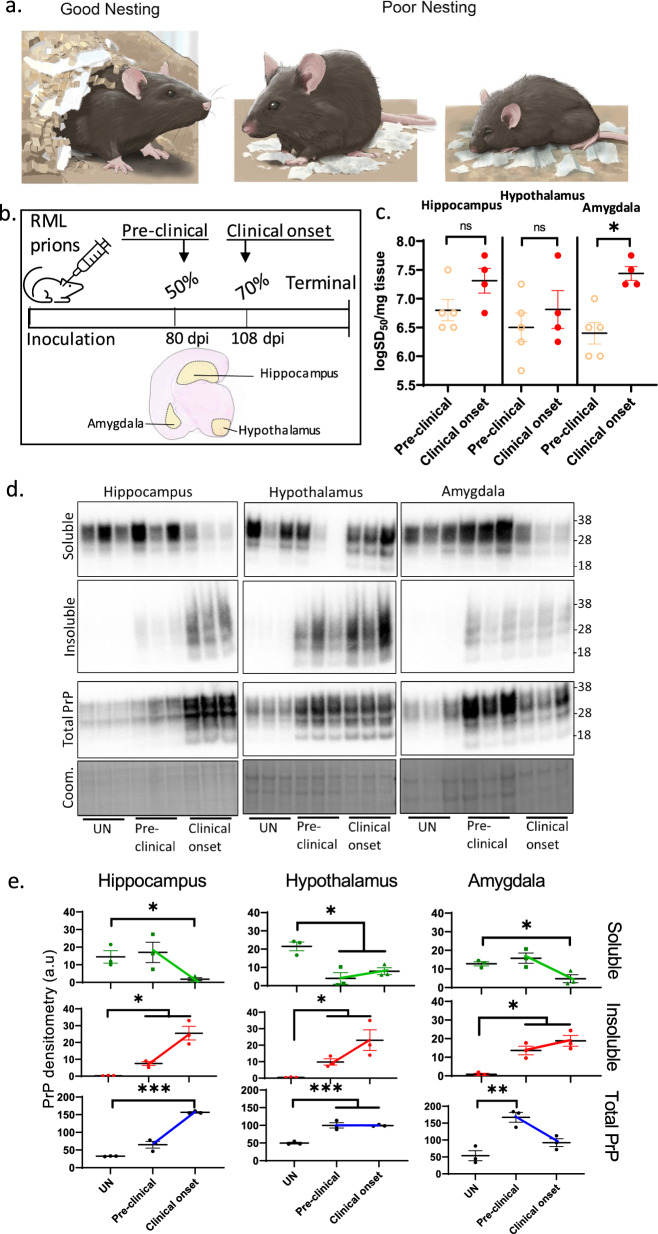
Prion propagation in the limbic regions during disease. **a** An illustration of reduced nesting and mood disorder-like symptoms in a mouse with prion disease (middle, right) compared with a normal healthy mouse (left). **b** A schematic diagram illustrating the timeline of the study, where weanling wild-type mice were inoculated with RML prions, and ex vivo fresh coronal slices of the limbic regions (hippocampus, hypothalamus, amygdala) were used for the assessments in this study at a pre-clinical stage or mid-incubation period (50% of the disease progression to terminal stage disease at ~ 80 days post-inoculation, dpi) and at clinical onset (~ 70% of disease progression or at ~ 108 dpi). **c** Prion seeding activity (logSD50) in the limbic regions at the pre-clinical and clinical onset (Unpaired Student’s t-test was used to compare prion seeding activity between two disease time points within a region). **d** Western blotting for PrP (6D11 antibody) after an ultracentrifugation separation of soluble from insoluble isoforms, relative to the total PrP, in the three limbic regions in samples from uninfected controls (UN) and the two disease time points. The bottom panels are Coomassie stains of total protein for the total PrP blots. Molecular weight markers are on the right. **e** The quantification of the blots in d. PrP at each time point was compared to the UN control by unpaired Student’s t-test. **c**, **e** Each dot represents a mouse, and data are presented as mean ± SEM. *p < 0.05, **p < 0.01, ***p < 0.001

Reduced nesting is one of the earliest clinical signs of PrD in mice (Fig. 1a) [2], starting at around 70% of terminal stage disease (TSD) or 108 days post-inoculation (dpi) in mice infected with RML prions. Such behavioral changes are associated with mood and depression, and linked to dysfunction of the limbic system. This system consists of various inner regions of the brain, including the hippocampus, hypothalamus, and amygdala. To determine the kinetics by which prions propagate in these regions, we inoculated mice expressing WT PrP^C^ with RML prions and harvested brains at two disease stages, the pre-clinical stage at 80 dpi (~ 50% TSD) where little vacuolation is observed, and the clinical onset at 108 dpi (~ 70% TSD) where vacuolation is seen moderately [8]; Fig. 1b, top panel). The hippocampus, hypothalamus, and amygdala were dissected out of fresh coronal brain sections to analyze prion biochemical properties during disease (Fig. 1b, bottom panel). All three brain regions contained prion seeds as measured by RT-QuIC assay, which appeared to increase as the disease progressed (Fig. 1c). To further classify prions biochemically, we analyzed the ratio of soluble to insoluble PrP species by sarkosyl insolubility assay. Total PrP was increased in infected samples relative to the uninfected controls (Fig. 1d, e). Thus, we normalized the soluble and insoluble PrP to the total amount to correct for the disease-associated increase in total PrP. Relative to the uninfected controls, the soluble PrP was reduced in all three regions at clinical onset, while it remained largely unaltered at the pre-clinical stage except for a decrease in the hypothalamus (Fig. 1d, e). The loss of soluble PrP supports a progressive propagation of insoluble prions in these limbic regions at the expense of soluble PrP species including PrP^C^. Further, NeuN level was not different from uninfected controls at the pre-clinical stage in all regions, becoming reduced at clinical onset only in the hypothalamus (see supplementary file 1). This suggests that the soluble PrP^C^ loss appears independent of neuronal loss. Overall, prion infection propagates within the limbic regions depositing insoluble prions, dysregulating PrP expression, and depleting soluble PrP levels.

### Basal synaptic transmission dysfunction at the pre-clinical-to-clinical disease transition

**Fig. 2:**
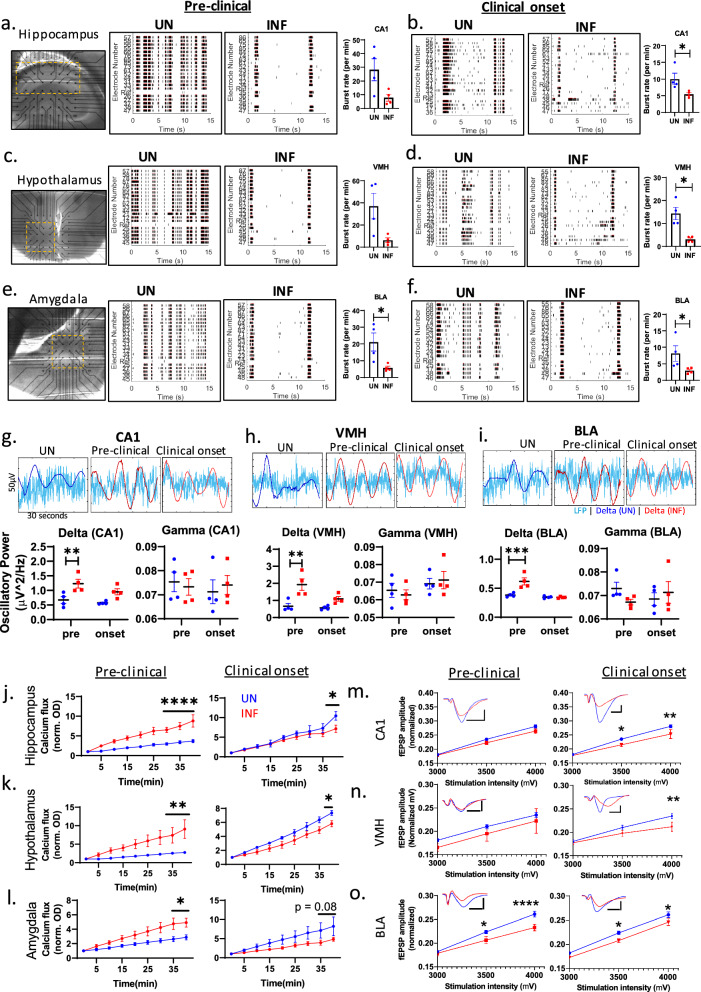
Dysfunction of basal neuronal activity. **a**–**f** Spontaneous synchronous neuronal firing in hippocampal CA1 (**a**, **b**), ventral medial hypothalamus (VMH; **c**, **d**), and basolateral amygdala (BLA; **e**, **f**) at the pre-clinical stage (**a**, **c**, **e**) and at clinical onset (**b**, **d**, **f**) measured by MEA (left panels of **a**, **c**, **e**; black dots are electrodes with the regions of interest marked by the dotted box). Raster plots (middle panels) present the neuronal firing (black dash) and burst (red line) with the quantification of burst rate (scatterplot on the right), comparing uninfected controls (UN) and infected mice (INF). See supplementary file 2 for representative burst traces. **g**–**i** Relative oscillatory powers of delta and gamma oscillations in UN versus INF at the pre-clinical stage and clinical onset in the CA1 (**g**), VMH (**h**), and BLA (**i**). Upper panels are representative traces of local field potentials (LFP; light blue) and delta waves from UN (dark blue) and INF (red). See supplementary file 3 for delta and gamma peak frequency. **j**–**l** Calcium flux analysis in the three limbic regions (hippocampus, hypothalamus, amygdala) at the pre-clinical stage and clinical onset (n = 3 per group). **m**–**o** Input–output curves measuring the magnitude of field excitatory post-synaptic potential (fEPSP) responses to increasing strength of stimuli to assess the impact of prion disease on the strength of synaptic transmission in the three limbic regions (CA1, VMH, BLA) at the pre-clinical stage (left panels; n = 8 for UN; n = 4 for IN) and clinical onset (right panels; n = 8 for UN; n = 4 for IN). Representative traces of fEPSPs are presented in each plot (vertical bars represent 100 µV and horizontal bars show 10 ms). Data were analyzed by unpaired Student’s t test (**a**–**i**) and Two-way ANOVA (assuming sphericity) with repeated measures and Sidak corrections for multiple comparisons. Data (biological replicates) are presented as mean ± SEM. *p < 0.05, **p < 0.01, ***p < 0.001, ****p < 0.0001

Neurodegeneration disrupts neuronal network connectivity. Our assessment of the spontaneous synchronized network activity showed a progressive decline in neuronal burst rate in the hippocampal CA1, ventromedial hypothalamus (VMH), and basolateral amygdala (BLA) of infected mice relative to uninfected controls. This started at the pre-clinical stage and reached statistical significance for all regions at clinical onset (Fig. 2a–f; supplementary file 2). Neuronal network decline was consistent with a significant increase in relative oscillatory powers and a significant decrease in peak frequency of delta waves in infected mice, which peaked before clinical onset (Fig. 2g–i; supplementary file 3). However, gamma oscillatory powers, at much smaller magnitudes than the delta waves, remained unaltered (Fig. 2g–i). The peak frequency of gamma oscillation was also unaltered (supplementary file 3). These data demonstrate that PrD induces slowing of neural oscillation at pre-clinical stage, as indicated by the enhanced oscillatory power of delta waves and the reduced fast neuronal population spiking, leading to the significant loss of neuronal network activity at clinical onset.

Synaptic transmission is calcium dependent. We assessed calcium influx in limbic regions at the pre-clinical stage and clinical onset. Relative to uninfected controls, the calcium influx was significantly increased in all three regions at the pre-clinical phase, and it became reduced at clinical onset (Fig. 2j–l). This suggests that the disease begins with dysregulating calcium influx at pre-clinical stage before overt disruptions at clinical onset, thus supporting the progressive slowing of neural oscillation and decline in spontaneous neuronal firing.

To further examine the efficacy of synaptic transmission, we stimulated these regions with increasing strength of electric stimuli (Fig. 2m–o). Relative to uninfected controls, the synaptic transmission was significantly disrupted in all regions at clinical onset, while only the BLA synaptic transmission was reduced at the pre-clinical stage (Fig. 2o). Overall, all three limbic regions exhibited an impairment of basal synaptic transmission, mostly at clinical onset, associated with deteriorating calcium flux.

### Exacerbated loss of synapses in limbic regions at the transition from pre-clinical to clinical disease

**Fig. 3:**
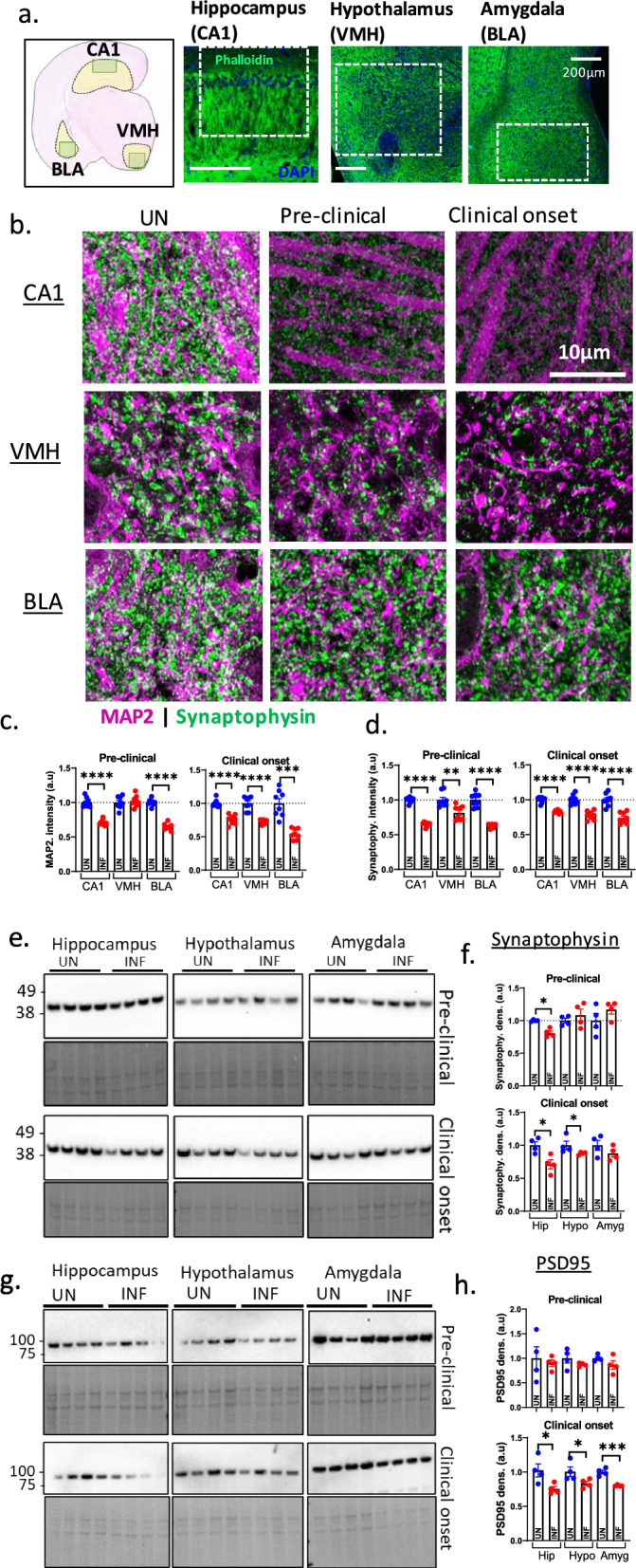
Synaptic changes in limbic regions during disease. **a** Subregions within the three limbic regions (schematic diagram), including the hippocampal CA1, ventral medial hypothalamus (VMH), and basolateral amygdala (BLA), were assessed by immunofluorescence (IF) labelling for synapses. The right panels display the subregions labeled with phalloidin (green), where the dotted boxes mark the specific regions assessed by higher magnification confocal microscopy. **b** Immunofluorescence assessment of total synapses by MAP2 and synaptophysin in the CA1, VMH, and BLA in uninfected controls (UN) relative to infected (INF) samples from the pre-clinical and clinical onset phases. **c**, **d** The quantification of MAP2 (**c**) and synaptophysin (**d**) in multiple fields of view of immunofluorescence images represented in **b**. Each dot represents a field of view. **e** Western blotting analysis of synaptophysin in the three brain regions at both time points with the lower panels showing the Coomassie stain for total protein as the loading control. **f** The quantification of synaptophysin normalized to the total protein. **g** Western blotting analysis of PSD95 in the three brain regions at both disease time points with the bottom panels showing Coomassie stain for loading control. **h** The quantification of PSD95 after normalizing to total protein. **c**, **d**, **f**, **h** Unpaired Student’s t-test was used to analyze the disease-related change in each marker relative to its respective UN control. Data (biological replicates) are presented as mean ± SEM. *p < 0.05, **p < 0.01, ***p < 0.001, ****p < 0.0001

Synaptic transmission depends on how many synapses are available. We assessed total synapses by immunofluorescence labeling of dendritic spine marker MAP2, and pre-synaptic protein synaptophysin in the hippocampal CA1, VMH, and BLA (Fig. 3a). The levels of post-synaptic spines detected by MAP2 appeared to diminish consistently in all three regions at clinical onset as compared with the uninfected controls (Fig. 3b, c). Similar disease-related synaptic loss was observed with synaptophysin labeling of pre-synapses at both time points (Fig. 3b, d). These immunofluorescence assessments suggest synaptic loss in these limbic regions during disease that is exacerbated at clinical onset. To confirm this, we quantified levels of synaptophysin and PSD95 (for post-synapses) by western blotting analyses of homogenates from hippocampus, hypothalamus, and amygdala. We observed a loss of synaptophysin in the hippocampus and hypothalamus at clinical disease, but no significant change in the amygdala (Fig. 3e, f). PSD95 became prominently reduced in all three regions at clinical onset, revealing a significant post-synapse loss (Fig. 3g, h). Overall, synaptic markers in these limbic regions are reduced following onset of disease, and this is likely associated with the basal synaptic dysfunction.

### Cytoskeletal damage in limbic regions is associated with clinical disease onset

**Fig. 4:**
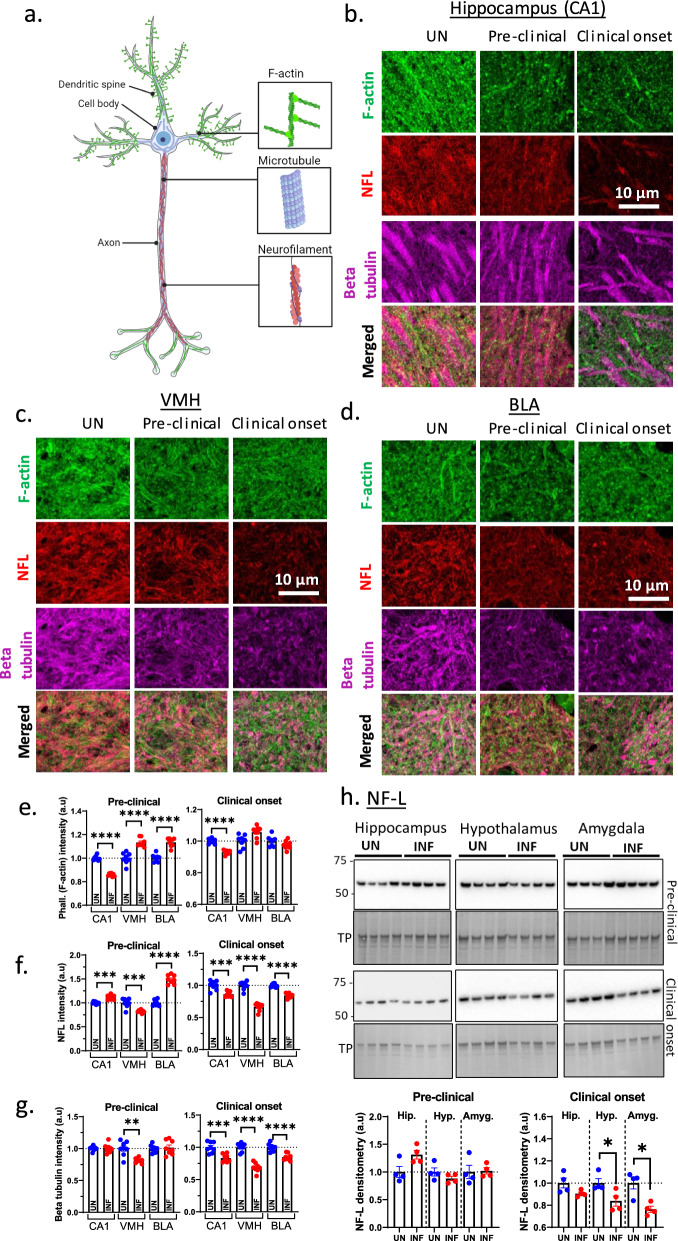
Cytoskeletal damage that may impede synaptic transmission in the limbic regions during prion disease. **a** A schematic diagram (Biorender) of a neuron showing the cytoskeletal markers analyzed, including filamentous actin (F-actin), microtubules (beta-tubulin), and neurofilament (NF-L). **b**–**d** Immunofluorescence analysis of the cytoskeletal markers in the hippocampal CA1, ventral medial hypothalamus (VMH), and basolateral amygdala (BLA) in uninfected controls (UN) versus infected mice at the pre-clinical stage and clinical onset. **e**–**g** The quantification of phalloidin for F-actin (**e**), NF-L (**f**), and beta tubulin (**g**) in multiple fields of view of immunofluorescence images represented in **b**–**d**. Each dot represents a field of view. **h** Western blotting analysis of NF-L in the hippocampus, hypothalamus, and amygdala (containing all subregions) at the pre-clinical time point and clinical onset. Coomassie stain for total protein (TP) was the loading control. Bottom panels show the quantifications of NF-L after normalizing to TP. Each dot represents a biological replicate. **e**–**h** Unpaired Student’s t-test was used to compare the level of each cytoskeletal marker between INF and UN control in each region. Data are presented as mean ± SEM. *p < 0.05, **p < 0.01, ***p < 0.001, ****p < 0.0001

Synapse morphology depends on cytoskeletal support provided by filamentous actin (F-actin), neurofilament, and microtubules (Fig. 4a). An assessment of these cytoskeletal components by immunofluorescence at two time points relative to uninfected controls showed a loss of F-actin in the CA1 at both time points. In contrast, the VMH and BLA showed increased F-actin level at the pre-clinical stage, but this was not changed by clinical onset (Fig. 4b–e). At the pre-clinical stage, NF-L was increased in the CA1 and BLA, but reduced in VMH. At clinical onset, all regions showed reduced NF-L (Fig. 4b–d, f). Beta tubulin was reduced in all regions at clinical onset (Fig. 4b–d, g). These data suggest that the neural cytoskeleton becomes damaged as the disease develops. To confirm this, we performed western blotting analyses of NF-L (Fig. 4e, f) to quantify the cytoskeletal loss and this showed a significant loss in the hypothalamus and amygdala at clinical onset. This loss did not reach statistical significance in the hippocampus, likely due to larger neurons with a bigger cytoskeletal NF-L structure that would take longer to degenerate. Overall, we observed evidence of cytoskeletal damage in all the limbic regions that appears to start at the pre-clinical stage and deteriorate further by clinical onset, thus suggesting a potential impact on synaptic plasticity.

### Altered neurotransmitter levels and synaptic release

**Fig. 5:**
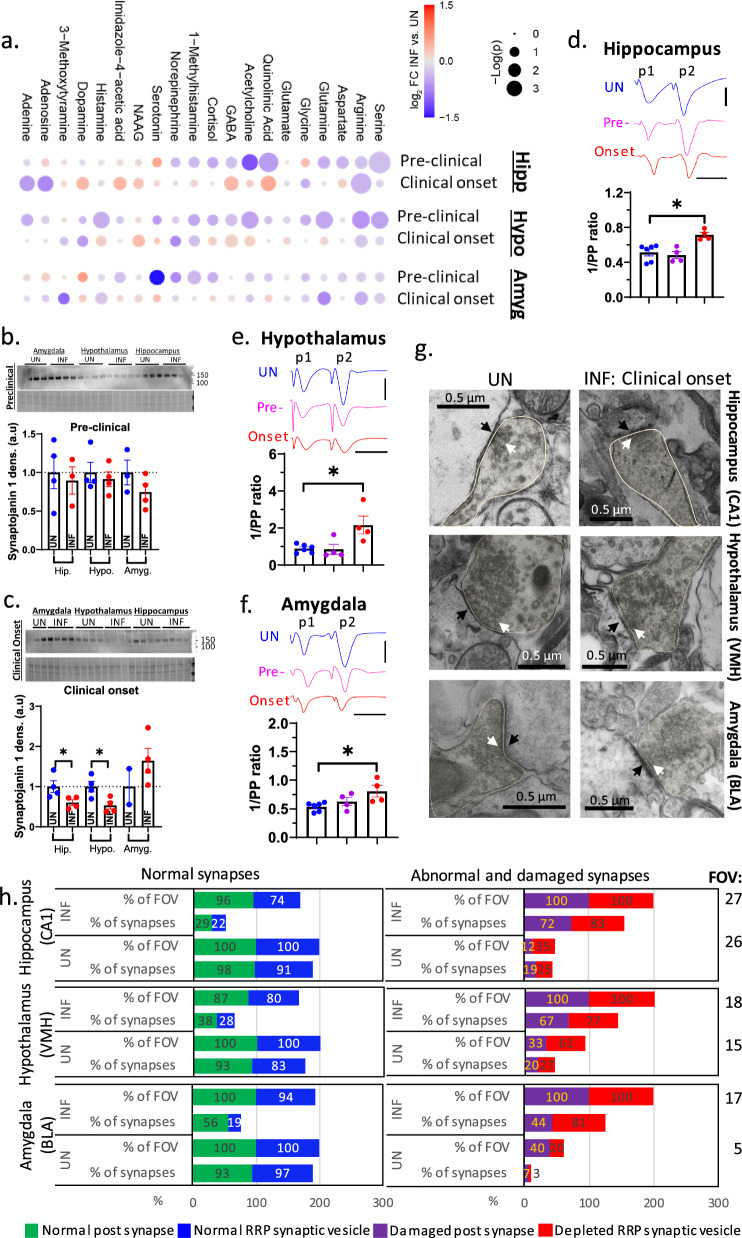
Neurotransmitter level and synaptic release during the disease transition from pre-clinical to clinical onset. **a** Bubble plots showing fold change of neurotransmitters in scrapie-infected (INF) hippocampus, hypothalamus, and amygdala (containing all subregions) at the pre-clinical disease and clinical onset relative to the age-matched uninfected (UN) controls (n = 4). **b**, **c** Western blotting analysis of synaptojanin 1 in each limbic region at the preclinical stage (**b**) and clinical onset (**c**) with the mean synaptojanin 1 level relative to its age-matched UN control compared by unpaired Student’s t test. **d**–**f** Paired-pulse (PP) ratios measured by paired-pulse test (See Methods) to estimate the probability of neurotransmitter release in hippocampal CA1 (**d**), ventral hypothalamus (**e**), and basolateral amygdala (**f**) of normal UN controls and INF mice at the two disease time points. Top panels are representative traces of field excitatory postsynaptic potentials (fEPSPs) evoked by the first pulse (p1) and the second pulse (p2). Vertical bar represents 100 µV and horizontal bar shows 20 ms. The mean 1/PP ratio was compared between UN and disease time points by Kruskal Wallis testing with Dunn’s correction for multiple comparisons. **g** TEM images in the hippocampus, hypothalamus, and amygdala in normal UN controls and INF mice with the pre-synaptic terminals are highlighted, harboring synaptic vesicles of neurotransmitters at the release sites (white arrows), adjacent the post-synaptic terminals (black arrow). **h** Bar graphs (by Microsoft Excel) displaying the scoring of TEM images or fields of view (FOV) based on the integrity of post-synapse and size of the readily releasable pool (RRP) of synaptic vesicles. This analysis (see Methods for more details) estimated levels of normal and damaged synapses and numbers of FOV with these phenotypes in uninfected and infected samples. The total FOV per group are listed on the right. Data in **b**–**f** (biological replicates) are presented as mean ± SEM. *p < 0.05

Synaptic transmission depends on the level of neurotransmitters and the efficacy of synaptic release. We measured levels of a panel of small molecule (non-peptide) neurotransmitters in all three limbic regions at pre-clinical and clinical onset relative to age-matched controls. A decrease or trending decrease relative to uninfected controls in certain neurotransmitters, including acetylcholine and GABA, was evident and conserved between brain regions at the pre-clinical stage. However, these differences either disappeared or began to trend upward, particularly in the amygdala, at clinical onset (Fig. 5a). Consistently, the level of synaptojanin 1, which is vital for synaptic vesicle recycling, was unchanged at pre-clinical stage, but it was reduced at clinical onset in the hippocampus and hypothalamus relative to uninfected controls (Fig. 5b, d; see IF images in supplementary file 4). These data suggest that disease onset is linked to changes in neurotransmitter levels and this may be related to neurotransmitter re-uptake and turnover.

We utilized the paired-pulse test, as described in the Methods, to estimate the probability of synaptic release (*pr*) from the readily releasable pool of synaptic vesicles. Relative to uninfected controls, the *pr* was unaltered at the pre-clinical stage, but it was significantly enhanced at clinical onset in all the limbic regions, demonstrating an increased propensity to release neurotransmitters (Fig. 5d–f). TEM images of pre-synaptic terminals (highlighted) showed a slightly lesser readily releasable pool of vesicles (depicted by the white arrow at the release site), with their morphology appearing unclear and deformed at clinical onset relative to uninfected controls in the three limbic regions (Fig. 5g). This was observed in all fields of view of infected tissues, with affected pre-synapses estimated to be ~ 83% in the hippocampus, ~ 77% in the hypothalamus, and ~ 81% in the amygdala (Fig. 5h). The lesser vesicles at the release sites suggests that they are depleted during disease. Interestingly, the amygdala at clinical onset accumulated more vesicles further away from the release site, which appeared to be the reserve pool of vesicles. This suggests that replenishment of the readily releasable pool is dysfunctional in the amygdala at clinical onset. Of note, the infected synaptic cleft (black arrow) appeared to have abnormal protein accumulation, which seemed more pronounced in the amygdala, compared with the uninfected controls (Fig. 5g). Taken together, the transition from preclinical to clinical disease involves a shift of neurotransmitter levels from being reduced to being increased in all three limbic regions, which was directly linked to an increase of synaptic release and depletion of the readily releasable pool of vesicles at the disease onset.

### Impairment of tetani-induced synaptic plasticity in limbic regions of scrapie infected WT mice

**Fig. 6:**
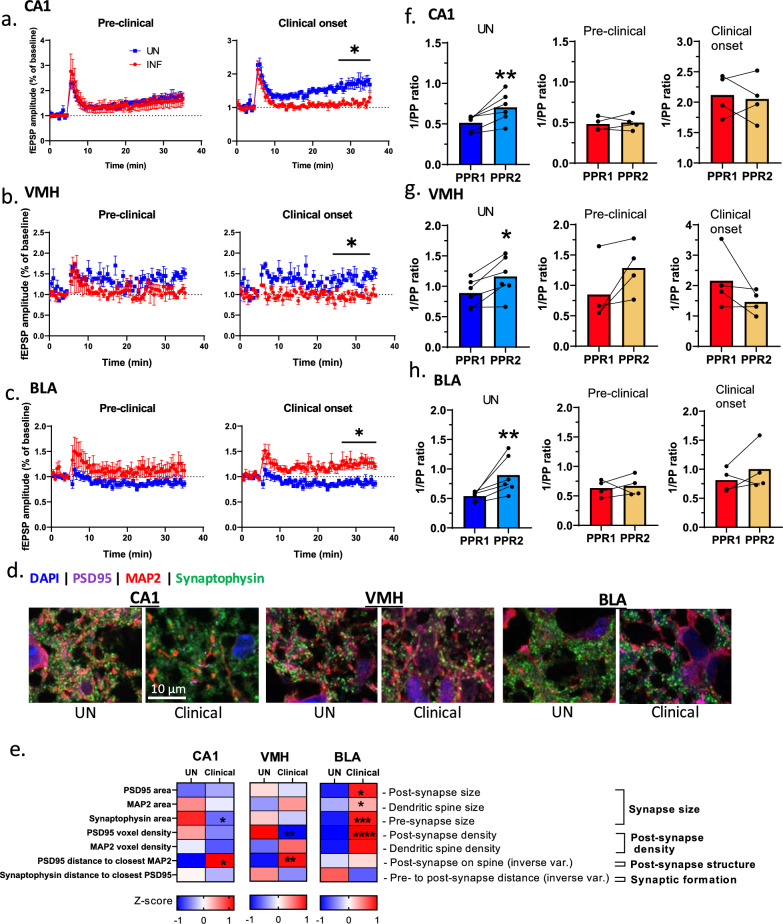
NMDA receptor-dependent long-term synaptic plasticity in limbic regions at the transition from pre-clinical to clinical disease. **a**–**c** Long-term synaptic plasticity induced by trains of high-frequency stimulation in the hippocampal CA1, ventral medial hypothalamus (VMH), and basolateral amygdala (BLA) at pre-clinical stage and clinical onset. Unpaired Student’s t-test was used to compare the mean field excitatory postsynaptic potential (fEPSP) of the last 10 min of the recording between uninfected controls (UN) and infected (INF) samples. Data are presented as mean ± SEM with n = 4 mice per group. **d** Representative immunofluorescence (IF) images of PSD95, MAP2, and synaptophysin in UN and INF CA1, VMH, and BLA after tetani induction of synaptic plasticity. **e** Estimates of various features of synapses by images (as represented in **d**) using segmentation approaches (n = 3/group). **f**–**h** The inverted paired pulse ratio (PPR) that estimates of the probability of neurotransmitter release before and after the synaptic plasticity induction (PPR1 and PPR2), indicating the release efficacy in the UN versus the INF samples at the pre-clinical stage and clinical onset. Each dot represents a mouse. Paired Student t-test was used to compare PPR1 and PPR2. Data (biological replicates) are presented as mean ± SEM. *p < 0.05, **p < 0.01, ***p < 0.001, ****p < 0.0001

Synapses are plastic and able to establish a prolonged change in reaction to stimuli. To assess the adaptive capacity of synapses (synaptic plasticity) in the limbic regions at the early stages of the disease, we used a tetanic induction protocol to induce long-term potentiation via activation of NMDA receptors, which are affected earlier in prion disease [28]. In uninfected controls, tetani induced a long-term potentiation in the hippocampal CA1 and VMH, while it inhibited long-term potentiation in the BLA. The opposite synaptic plasticity was observed when NMDA receptors in uninfected controls were pharmacologically blocked with D-AP5 (see supplementary file 5), demonstrating the dependence of the tetani-induced plasticity on these receptors. Relative to uninfected controls, long-term synaptic plasticity in the three limbic regions was not statistically changed at the pre-clinical stage. However, all regions showed dysfunctional synaptic plasticity at clinical onset, whereby the hippocampal CA1 and VMH expressed a reduced long-term potentiation while the BLA potentiated (Fig. 6a–c). This abnormal BLA potentiation was consistent with a significant increase of NMDA receptors, as measured by NR1, relative to uninfected controls, indicating that the disease interferes with the mechanisms that regulate protein levels of NMDA receptors (see supplementary file 6). This increase was not observed in the other two regions. Similar changes in synaptic markers were observed at clinical onset, where MAP2, PSD95, and Synaptophysin were increased in the BLA, while reduced in the hippocampal CA1 and VMH (see supplementary file 7). The increased levels of synaptic markers in the BLA appeared to be due to larger synapses, although fewer in number than in the uninfected controls, as estimated by the size and quantity of synaptophysin puncta by immunofluorescence (Fig. 6d, e; see supplementary file 8). These larger synapses were not evident in the other regions.

Further, the dysfunctional synaptic plasticity was consistent with the inability of synapses to increase their *pr* in response to tetani in all three regions at either time point. This was demonstrated by comparing the PPRs measured before and after tetani, whereby normal controls showed a significant increase in inverted PPRs post-tetani while the infected samples failed to exhibit such increase (Fig. 6f–h). Thus, the disease interferes with mechanisms that increase synaptic release to maintain long lasting plasticity. Overall, disease-related changes of synapses directly contribute to abnormal synaptic plasticity in the limbic regions at clinical onset. Such changes include dysfunctional neurotransmitter release and abnormal cytoskeleton remodeling in all three regions and a dysregulated NMDA receptor level specifically in the amygdala.

## Discussion

One interesting aspect of PrDs is the selective vulnerability of different areas of the brain. Here we investigated how PrD affects the limbic system by assessing the synaptic transmission in three major limbic regions essential for cognition and behavior: the hippocampus, hypothalamus, and amygdala. We found significantly impaired basal synaptic transmission in all regions, specifically in the hippocampal CA1, VMH, and BLA at around 70% of the disease incubation period, which is when the earliest clinical signs can be observed in these mice. However, the regions did not show identical changes in response to infection. A noteworthy observation is that the amygdala seems affected by disease progression differently from the hippocampus and hypothalamus. This is consistent with individual brain regions being attacked differently in distinct PrDs, likely contributing to the broad range of symptomology that occurs in humans.

We found evidence that neuronal dysfunction and synaptic changes begin before neuronal loss becomes detectable at early clinical onset. Dysfunctions included dysregulated calcium influx and slowing neural oscillation in all limbic regions, which suggest excitotoxicity associated with an imbalance of excitation-to-inhibition. By 70% of disease progression these changes led to a substantial neuronal dysfunction that corresponded with the onset of reduced nesting and burrowing behaviors in scrapie-infected mice, which are rodent signs of disease [2, 29]. Previous studies have also found a link between CA1 long-term potentiation impairment and clinical onset in mice [13], that dysfunction is associated with PrP^Sc^ deposition within the limbic system [29], and that synaptic loss precedes neuronal loss and behavioral defects [30, 31]. Interestingly, herein, neurotransmitter levels were generally reduced pre-clinically, suggesting either a down-regulation of their synthesis and storage or an excessive release for neuronal function with potentially impaired up-take. However, by the 70% time point, a shift in certain neurotransmitters to increased levels was observed in all three regions. It is feasible that down regulation of neurotransmitter levels is triggered by excitotoxic signaling and that, as this progresses to neuronal dysfunction and death, compensatory mechanisms fail to maintain excitatory signaling.

Previous research has found that around mid-disease, 22L infected mice show increased phosphorylated AMPA receptors and reduced mGLUR5 dimers [32]. This was linked with activity regulated cytoskeleton-associated protein (Arc/Arg3.1) response, which was seen to be significantly up-regulated earlier in the incubation period. Indeed, neuronal dysfunction may be caused by changes in synaptic structure and composition as supported by the cytoskeletal breakdown found both herein and in previous studies (reviewed in [33, 34]). Sigurdson and colleagues also found that AMPA receptor derangement was influenced by the functioning of the ESCRT-0 complex, which itself requires the cytoskeleton to traffic endosomes [35].

An inability to traffic damaged receptors away from the synapse is certainly likely to result in neuronal network dysfunction and a loss of PrP function itself might also be involved. Various hypotheses have been formulated that neuronal dysfunction and loss may be directly due to changes in or loss of PrP^C^ function during disease progression. A progressive loss of PrP^C^ in the whole brain of scrapie-infected mice has been observed after the mid-incubation period, and this was related to accumulation of PrP^Sc^ [36]. Our results support that reduction in the availability of soluble PrP^C^ correlates with the loss of normal function. PrP^C^ has been reported to have a neuroprotective role against oxidative damage [37], and the loss of PrP^C^, with accumulation of toxic PrP^Sc^, have been shown to correlate with heightened oxidative stress during disease [38, 39]. When under oxidative stress, an absence of GPI-anchored PrP^C^ causes neurons to become hyperexcitable, leading to significant degeneration of synapses and breakdown of the cytoskeleton associated with oxidatively modified actin [37]. Our observation of an increased calcium influx in all three limbic regions at the mid incubation period before clinical onset suggests some degree of dysregulated neuronal excitability, which may be stabilized by PrP^C^ until its levels begin to decline. This early dysregulation is consistent with the detection of synaptotoxic prion as well as abnormal astrogliosis (see SI9) and oxidative stress in the brains of mice at clinical disease [38, 40, 41].

Stabilization of actin is important for synaptic health and function. Dendritic spines contain high concentrations of actin whilst mostly excluding other microtubule components [42]. Actin stability is regulated by glutamate receptor function, with NMDA stimulation thought to stimulate spine formation, which is then stabilized by AMPA activation [43]. Likewise, disruption of actin causes pronounced changes in dendritic spine electrical activity suggesting feedback is important in both directions [44]. Spine shrinkage and weakening has been associated with various neurodegenerative diseases [34]. The abnormal synaptic plasticity at clinical onset in all three limbic regions is consistent with a lack of synaptic maintenance, a vital compensatory mechanism to ensure healthy neurons [45]. Furthermore, previous studies have found that addition of PrP^Sc^ to murine neuronal cultures caused rapid retraction or collapse of the dendritic spines [46, 47]. In our study, by 70% of the incubation period, the hippocampal and hypothalamic neurons showed impaired long-term potentiation, likely due to the observed synaptic changes and despite increased neurotransmitter production.

In the amygdala, the disease appeared to increase the size of synapses, suggesting that the pruning machinery could only remove smaller synapses but was insufficient to prune larger synapses. Consistent with our finding of poor synaptic maintenance, it has been shown that synaptic morphological abnormality associated with PrD is due to a neuron-autonomous event that is independent of glia, in which the dendritic spine wraps around degenerating pre-synapses, thus causing enlarged synapses and synaptic loss [48]. That the amygdala behaved distinctly from the other limbic regions tested herein is an indication that its function may be disrupted by prion infection differently. One potentially relevant finding is that copper has been linked with amygdala function [49]. PrP^C^ is known to bind copper [50, 51] and PrP^C^ has been shown to modulate NMDA receptor function in association with this copper binding [52]. Scrapie-infected mice have also been shown to gain more dendric spines at an early clinical disease stage before progressively losing them due to the collapse of synaptic terminals, which appeared independent of microglia and their synaptic pruning role [48, 53]. Thus, the amygdala may later also lose even the enlarged synapses.

The synaptic degeneration and transmission deficits we observed in the hypothalamus at clinical onset are consistent with the disease causing endocrine system-related abnormalities, including disrupted sleep–wake cycle [54, 55] (reviewed in [56]), heightened aggressive behavior (reviewed in [7]), and increased fluid and glucose intake [57]. Despite the high glucose intake at the clinical stage, RML PrD prevents normal weight gain and causes weight loss in mice [2], suggesting a dysfunctional hypothalamic control of food intake and a significant abnormality of energy metabolism. For in-depth mechanistic insight into this abnormal food intake, further studies could assess the production of the ‘hunger hormone’ Ghrelin and Leptin in PrD. The high fluid intake is linked to scrapie-infected mice exhibiting increased bladder weight [5] and urinary incontinence [58], a clinical sign reported in human CJD [59], which may be due to a dysfunction of a nerve circuitry associated with the hypothalamus (reviewed in [60]).

There are limitations to our study. (i) The estrous cycle in female mice used in this study might influence the phenotypes we observed. Although female mice in certain mouse backgrounds, including C57, have been shown to have shorter disease incubation periods than male mice, the brain pathology at terminal disease was not different between genders, particularly in the hypothalamus that involves estrous cycle regulation [61]. However, we acknowledge the estrous cycle may contribute to the early synaptic dysfunction in the hypothalamus. (ii) The current study lacks information about how prions spread from the inoculation site (parietal area) to the limbic regions of interest, which could potentially influence the kinetics of synaptic dysfunction that we report here. A future study to inoculate with prions at more distant brain regions from the limbic area will address this concern. (iii) The paired-pulse ratio used to estimate synaptic release might also be influenced by the sensitivity of post-synapses and the efficiency of releasable pool replenishment from the reserve pool. However, our conclusion is based on a crude estimate of synaptic release, which will benefit from a future well-controlled study with appropriate techniques such as patch clamp and optogenetics to accurately measure synaptic release in individual neurons. (iv) Our amygdala electrophysiology recording was not specific to any of the pathways known to associate with the amygdala, such as the cortical and thalamic inputs [62]. Our research on early-stage prion disease will benefit from future studies designed to interrogate various brain circuits and how the disease affects various aspects of neural oscillations, such as spike-phase coupling, as reported in mouse models of Alzheimer’s disease [63, 64].

## Conclusions

Here, we report evidence that PrD disrupts synaptic transmission and plasticity in the hippocampal CA1, ventral medial hypothalamus, and basolateral amygdala at an early stage of clinical onset in scrapie-infected mice. Despite commonalities in the temporal onset of dysfunction, the different limbic regions showed several region-specific responses to the infection including changes in synapse structure and neurotransmitter levels. Our observations directly correlate with the early onset of reduced nesting in these mice, representing psychiatric symptoms associated with the disease in humans. Therapeutic drugs that alleviate these early neuronal dysfunctions may significantly delay clinical onset.

## Supplementary Information

Additional file 1.

Additional file 2.

Additional file 3.

Additional file 4.

## Data Availability

The datasets used and/or analyzed during the current study are available from the corresponding author upon reasonable request.

## Abbreviations

aCSF: Artificial cerebrospinal fluid
AMPA: α-Amino-3-hydroxy-5-methyl-4-isoxazolepropionic acid
BLA: Basolateral amygdala
BSE: Bovine spongiform encephalopathy
CA1: Cornus smmonis 1
CJD: Creutzfeldt Jakob disease
CWD: Chronic wasting disease
fEPSP: Field excitatory postsynaptic potential
GABA: Gamma-aminobutyric acid
MRI: Magnetic resonance imaging
NMDA: *N*-Methyl-d-aspartate
PP: Paired pulse
PPR: Paired pulse ratio
pr: Probability of release
PrD: Prion diseases
PrPC: Normal prion protein
PrPSc: Prions
PSD: Postsynaptic density
RML prions: Rocky mountain laboratories prion strain
RT-QuIC: Real-time quaking-induced conversion
TSD: Terminal-stage disease
VMH: Ventral medial hypothalamus
