# Supplementary material for: Limbic system synaptic dysfunctions associated with prion disease onset
Source: Acta Neuropathol Commun. 2024 Dec 20;12:192. doi: 10.1186/s40478-024-01905-w (PMC11662616; doi:10.1186/s40478-024-01905-w)

Uncropped blots

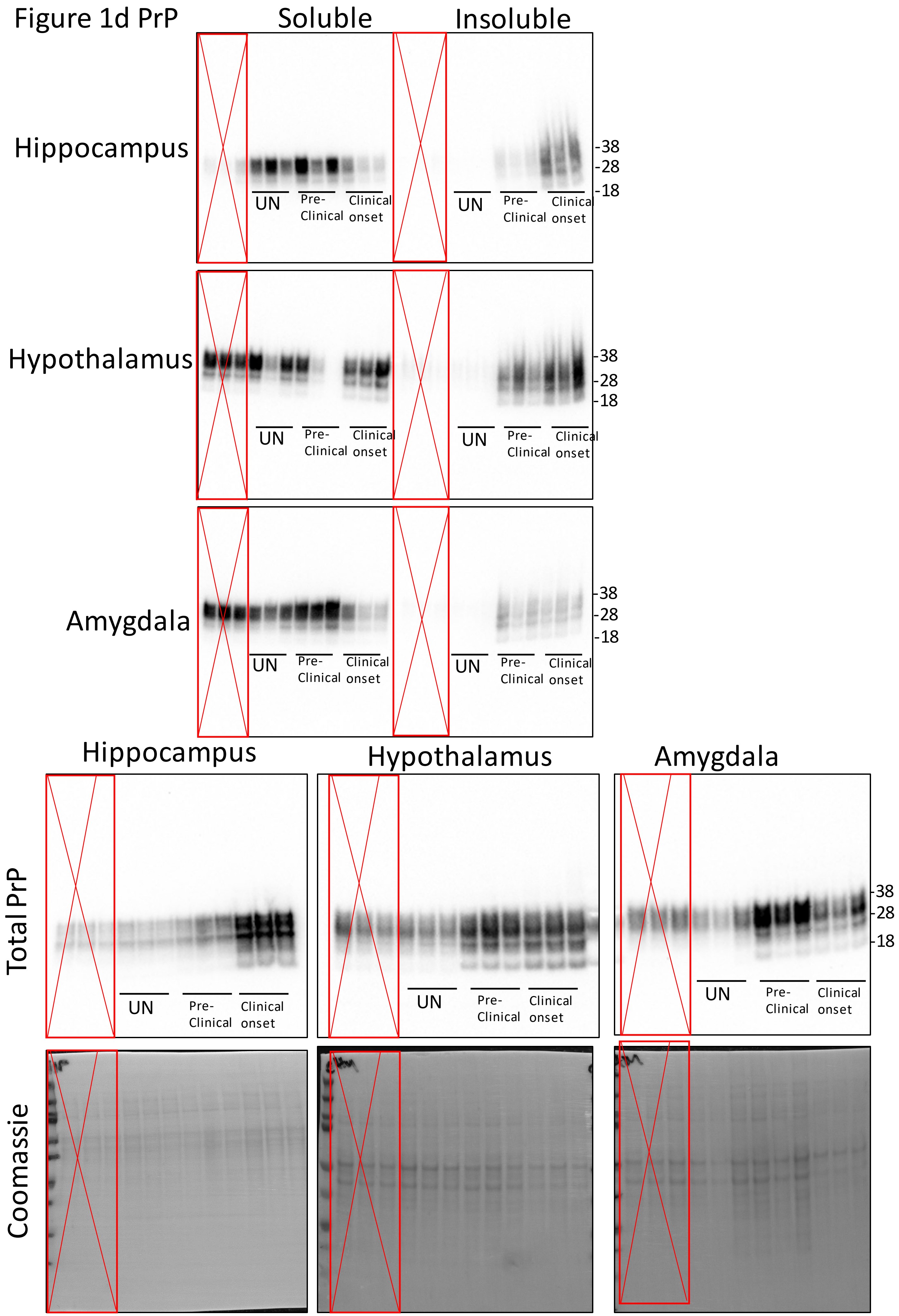

Figure 1d PrP
Soluble
Insoluble
Hippocampus
Hypothalamus
Amygdala
UN
Pre-Clinical
Clinical onset
38
28
18
Total PrP
Coomassie

Figure 3 e Synaptophysin

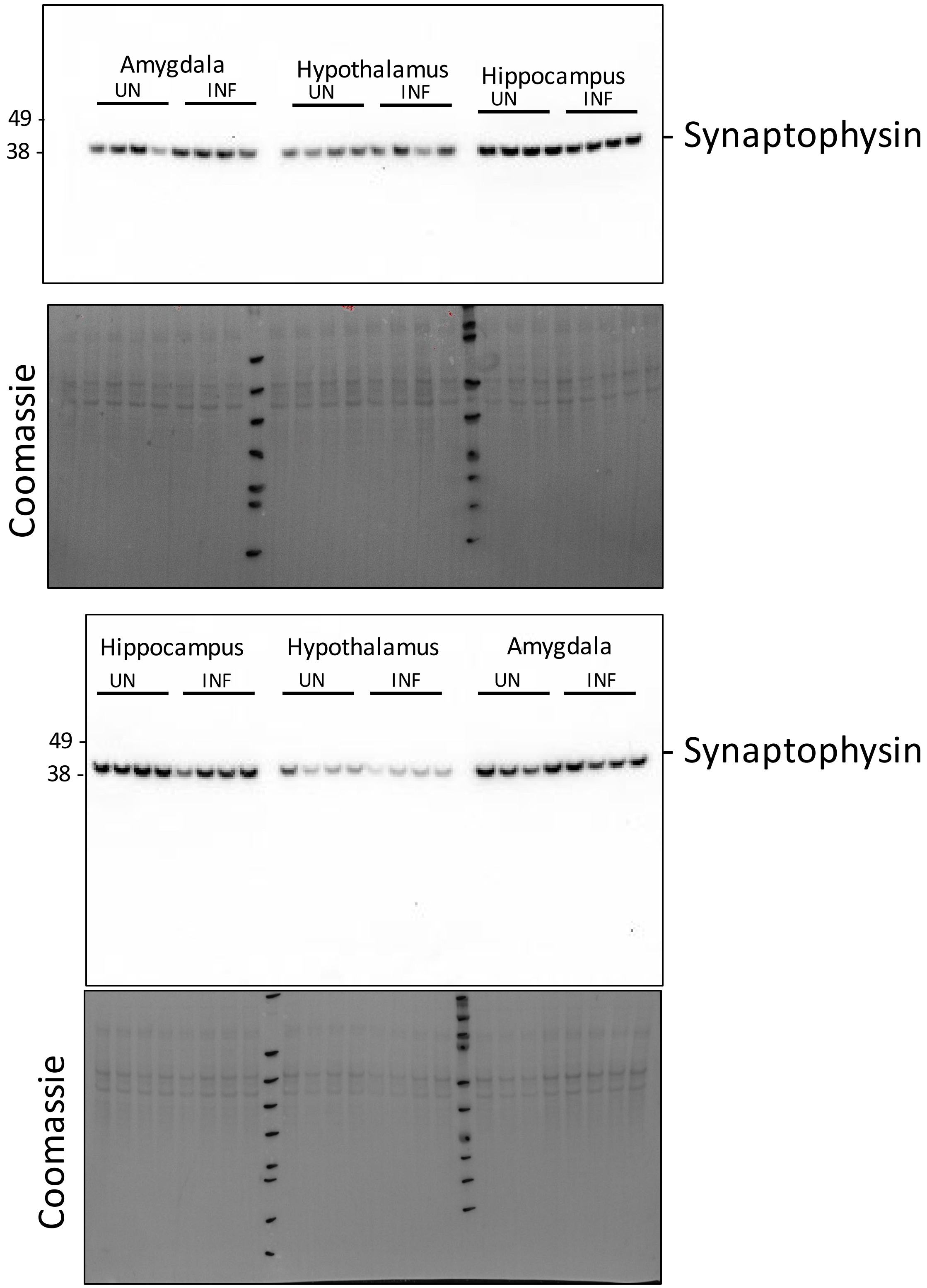

Figure 3 g PSD95

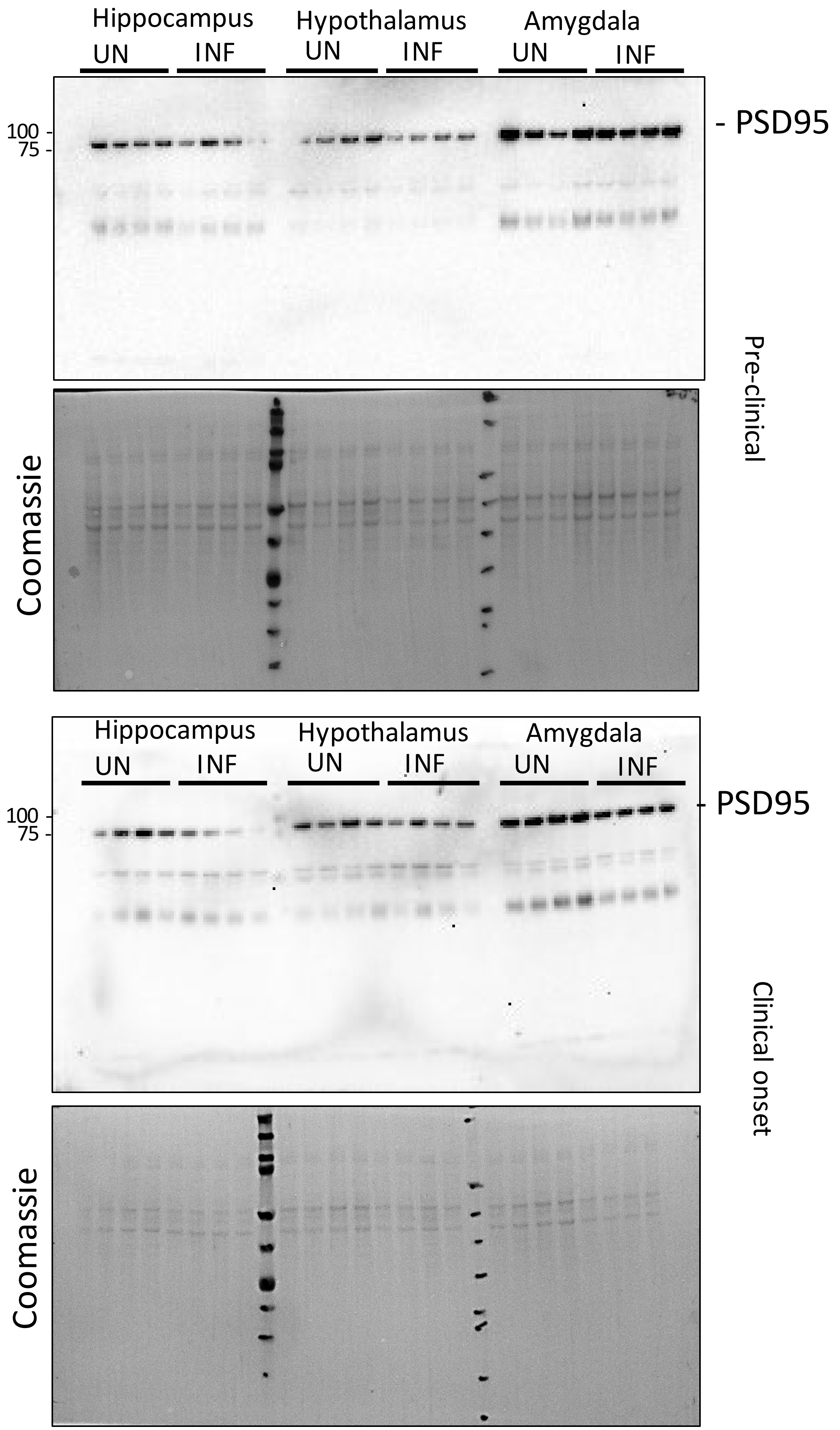

Figure 4 e NFL

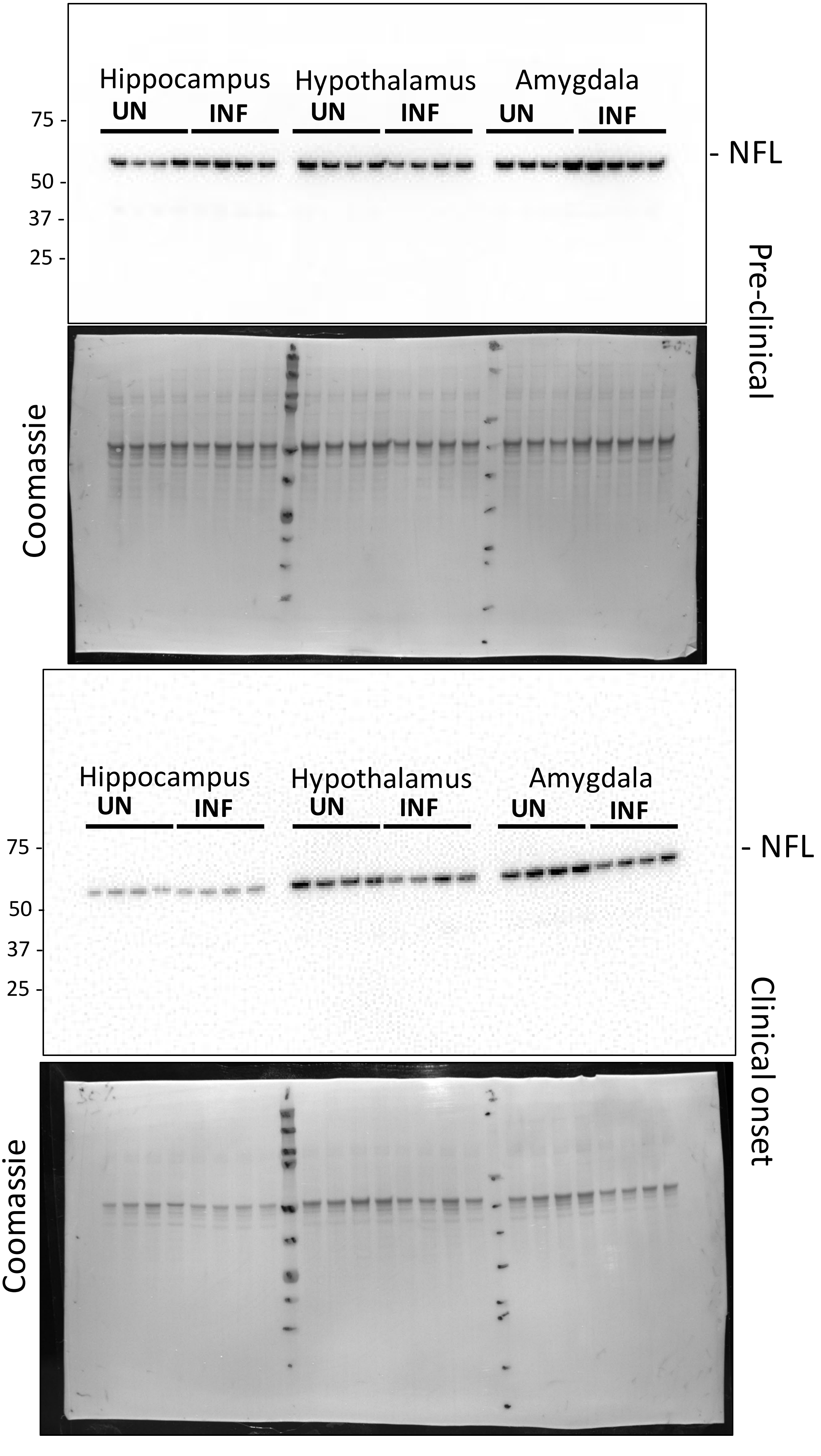

SI 2: Western blotting for NeuN

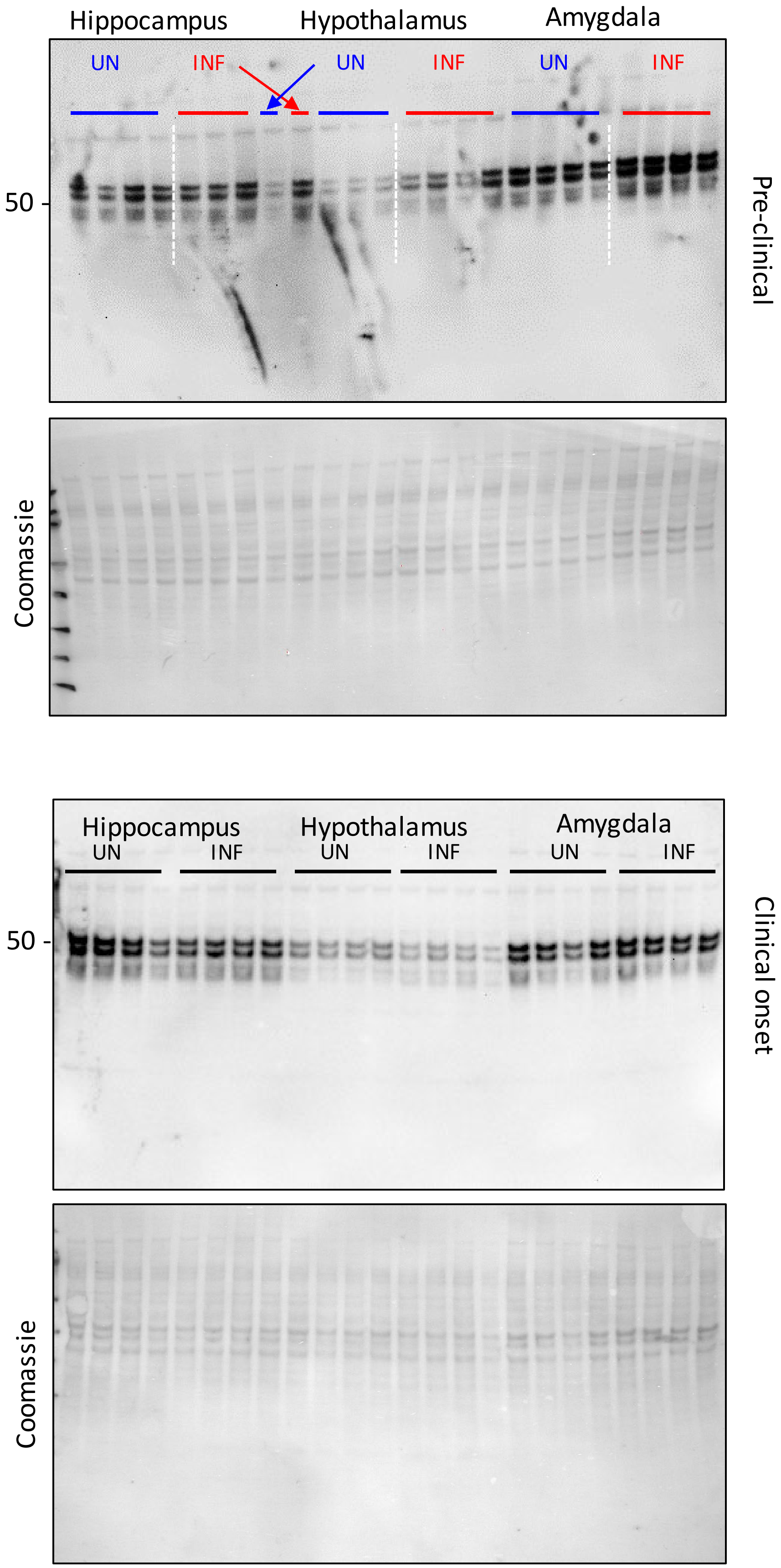

SI 8

GFAP level measuring astrogliosis

Pre-clinical

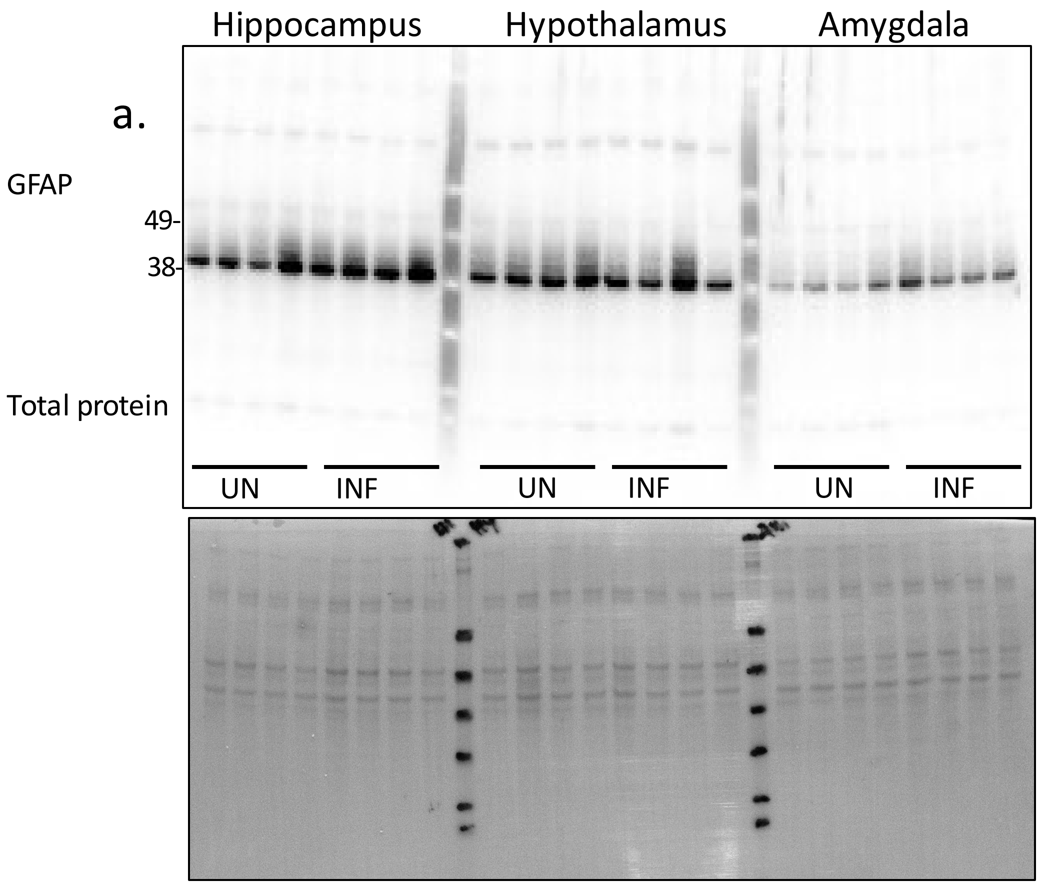

Clinical onset

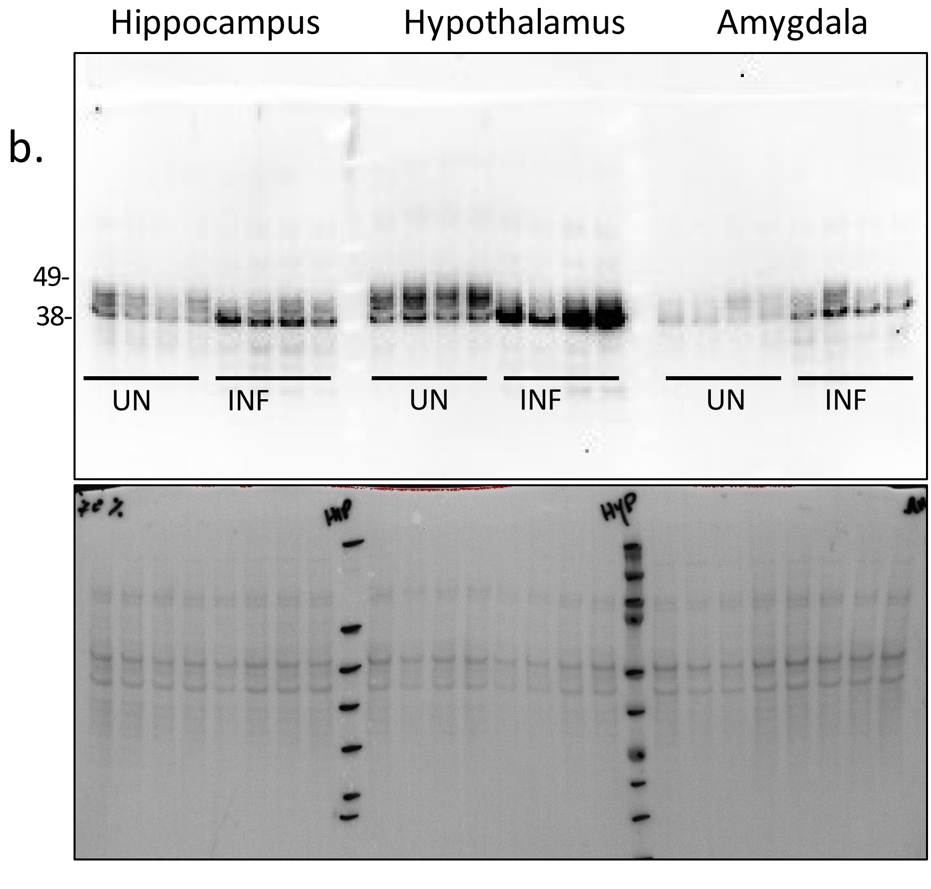

Supplement: Supplementary file 1 — Additional file 1. [file 40478_2024_1905_MOESM1_ESM.pdf]
