# Supplementary material for: Limbic system synaptic dysfunctions associated with prion disease onset
Source: Acta Neuropathol Commun. 2024 Dec 20;12:192. doi: 10.1186/s40478-024-01905-w (PMC11662616; doi:10.1186/s40478-024-01905-w)
Supplement: Supplementary file 2 — Additional file 2. [file 40478_2024_1905_MOESM2_ESM.pdf]

# **Additional file 1:** Antibodies

| Antibodies | From | Catalog # | Use (Dilutions) | Host species | Secondary antibodies for WB (Dilutions) | Secondary antibodies for IF (Dilutions) |
|---|---|---|---|---|---|---|
| **6D11** | BioLegend | 808001 | WB (1:5K) | Ms | Gt anti Ms HRP (1:5K) | -- |
| **MAP2** | SynapticSystems | 188006 | IF(1:500) | Ch | -- | Gt anti Ch Alexa Fluor 647 (1:500) |
| **Synaptophysin** | ABCAM | ab8049 | WB (1:5K)/IF(1:200) | Ms | Gt anti Ms HRP (1:5K) | Gt anti Ms Alexa Fluor 488 (1:500) |
| **PSD95** | ABCAM | ab18258 | WB(1:5K)/IF(1:200) | Rb | Gt anti Rb HRP (1:5K) | Gt anti Rb Alexa Fluor 488 (1:500) |
| **Phalloidin** | Invitrogen | A12379 | IF(1:500) | -- | -- | -- |
| **NF-L** | Invitrogen | 13-0400 | WB(1:5K)/IF(1:200) | Ms | Gt anti Ms HRP (1:5K) | Gt anti Ms Alexa Fluor 568 (1:500) |
| **Beta tubulin** | ABCAM | ab6046 | IF(1:200) | Rb | -- | Gt anti Ms Alexa Fluor 647 (1:500) |
| **Synaptojanin 1** | ABCAM | ab308136 | IF(1:100)/WB(1:1K) | Rb | Gt anti Rb HRP (1:5K) | Gt anti Rb Alexa Fluor 488 (1:500) |
| **NeuN** | ABCAM | ab177487 | WB(1:2K) | Rb | Gt anti Rb HRP (1:5K) | -- |

## **Additional file 2:** Western blotting for NeuN

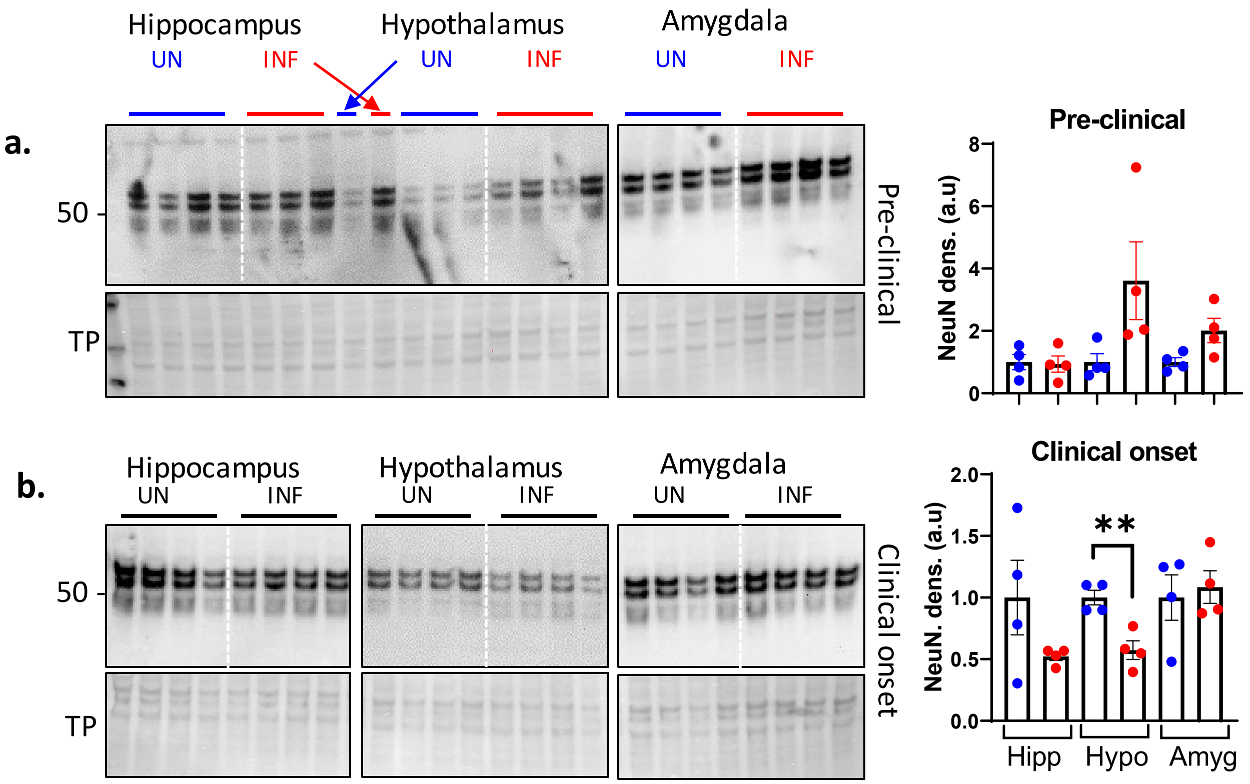


Western blotting analysis of NeuN level in the 3 limbic regions at pre-clinical stage(a) and clinical onset (b). Right panels are the quantifications comparing infected (INF) samples to age-matched uninfected (UN) controls by Unpaired Student's t test with Welch's correction. Data are presented as mean ± SEM. **p<0.01

## **Additional file 3:** Synaptojanin immunofluorescence analysis

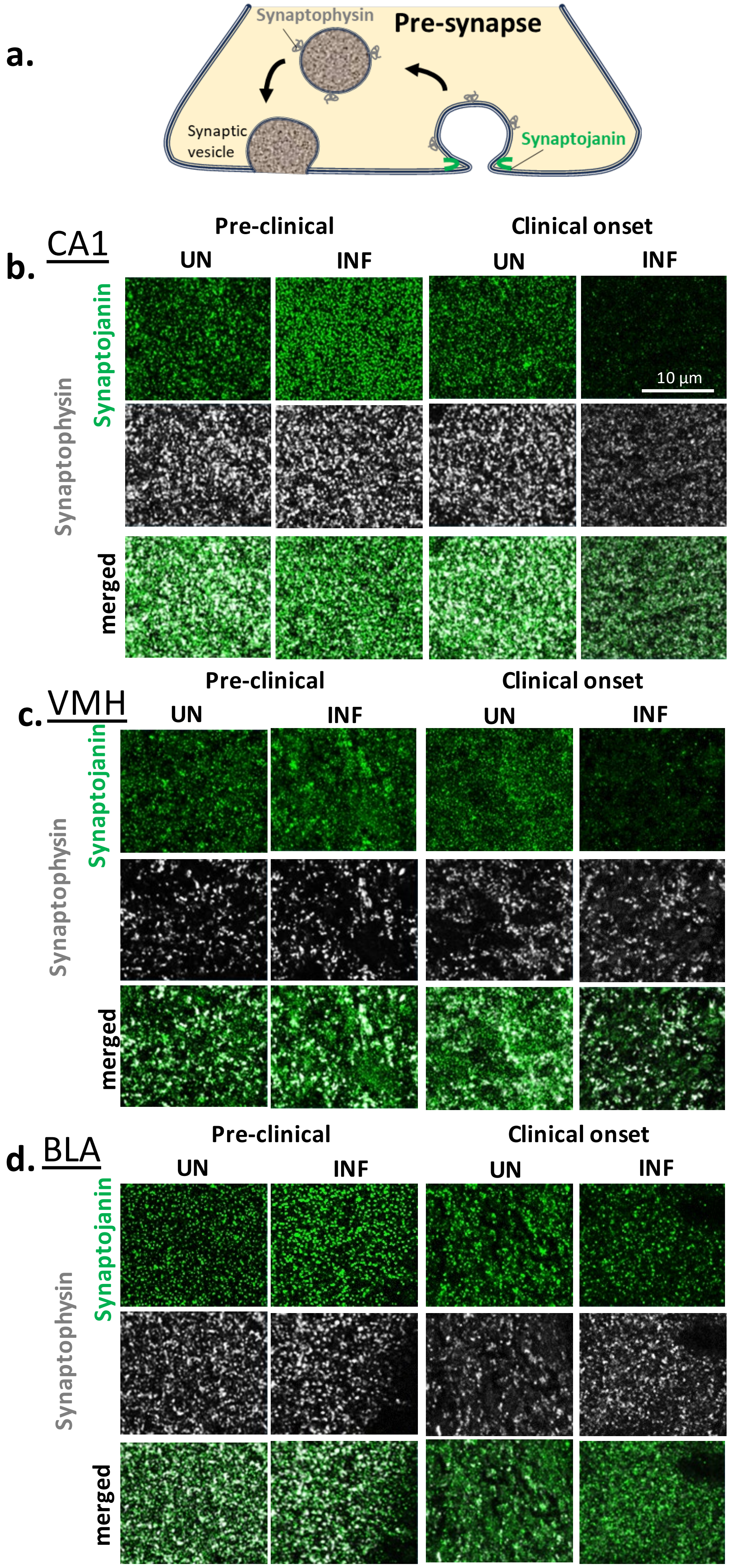


**a.** A schematic diagram of pre-synapse terminal with synaptophysin and synaptojanin localization.

**b-d.** Immunofluorescence analysis of synaptophysin and synaptojanin in the hippocampal CA1 (b), VMH (c), and BLA (d) at the pre-clinical stage and clinical onset relative to the age-matched uninfected controls.

**Additional file 4:** Synaptojanin analysis by western blotting

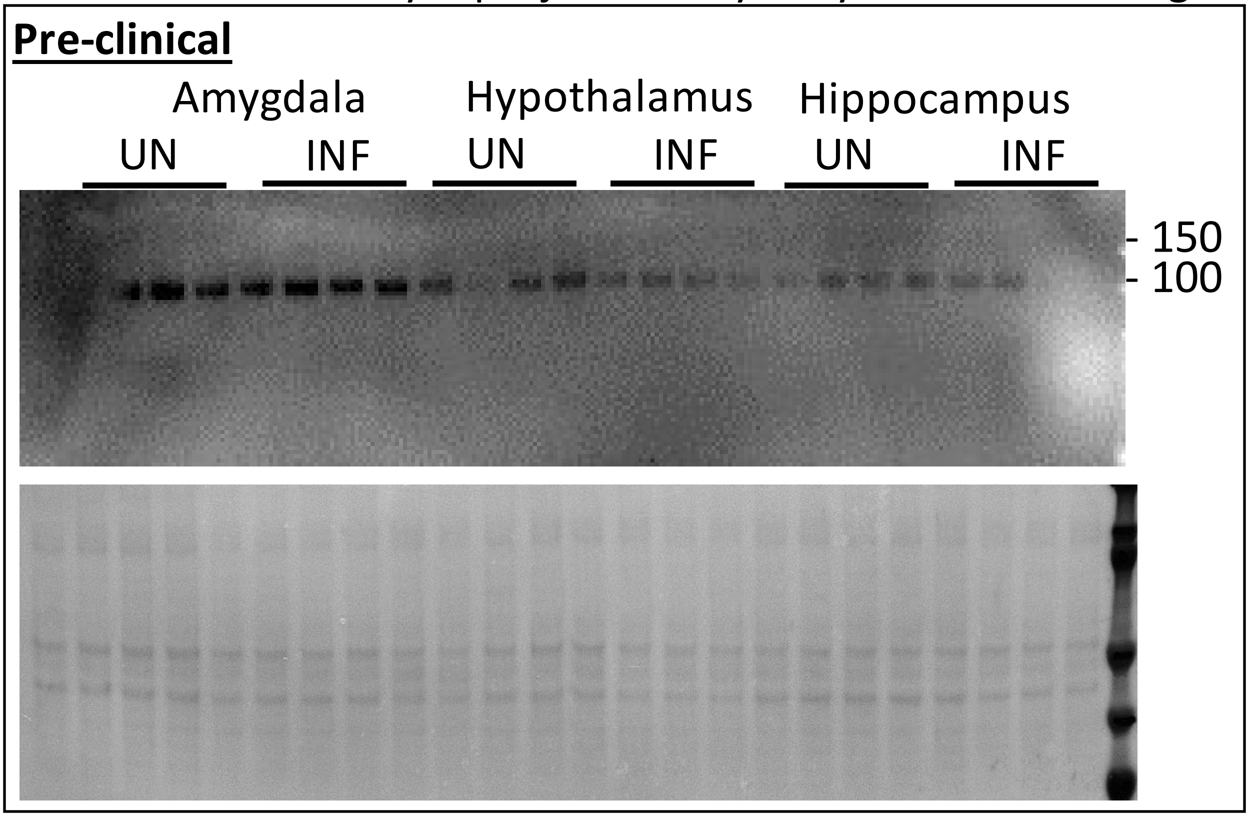


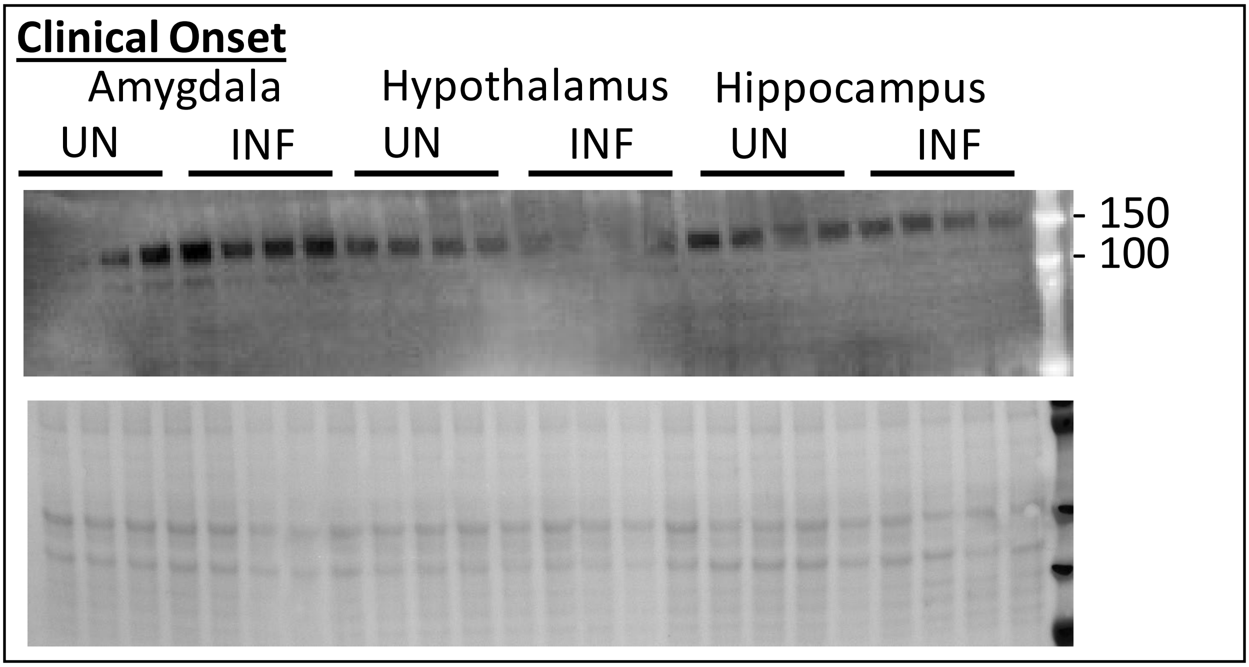


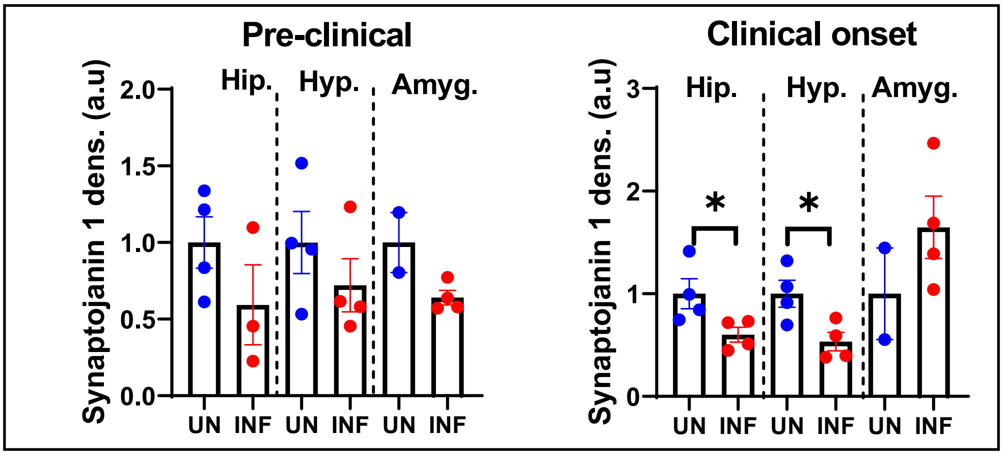


Western blotting analysis of synpatojanin 1 levels (~150KDa band) in the three limbic regions at the pre-clinical stage (top panel) and clinical onset (middle panel) relative to the uninfected age-matched controls. The lower panel shows the quantifications of the blots after normalizing to the total protein (TP) by Coomassie stain. Each dot represents a biological replicate (mouse). Data are presented as mean± SEM. *P<0.05

**Additional file 5:** TEM image analysis at clinical onset

## Normal healthy synapses

| | UN | | INF | | |
|---|---|---|---|---|---|
| Phenotypes: | Normal Post synapse | Normal synaptic vesicles | Normal Post synapse | Normal synaptic vesicles | |
| Hip. fields of view with phenotypes | 26 | 26 | 26 | 20 | Hippocampus |
| Total fields of view in Hip | 26 | 26 | 27 | 27 | |
| % of synapses with phenotypes | 98 | 91 | 29 | 22 | |
| % of fields of view with phenotypes | 100 | 100 | 96 | 74 | |
| Amy. fields of view with phenotypes | 5 | 5 | 17 | 16 | Amygdala |
| Total fields of view in Amy | 5 | 5 | 17 | 17 | |
| % of synapses with phenotypes | 93 | 97 | 56 | 19 | |
| % of fields of view with phenotypes | 100 | 100 | 100 | 94 | |
| Hyp. fields of view with phenotypes | 18 | 18 | 13 | 12 | Hypothalamus |
| Total fields of view in Hyp | 18 | 18 | 15 | 15 | |
| % of synapses with phenotypes | 93 | 83 | 38 | 28 | |
| % of fields of view with phenotypes | 100 | 100 | 87 | 80 | |

## Abnormal/damaged synapses

| | UN | | INF | | |
|---|---|---|---|---|---|
| Phenotypes: | Damaged post-synapse | Depleted synaptic vesicles | Damaged post-synapse | Depleted synaptic vesicles | |
| Hip. fields of view with phenotypes | 3 | 9 | 27 | 27 | Hippocampus |
| Total fields of view in Hip | 26 | 26 | 27 | 27 | |
| % of synapses with phenotypes | 19 | 25 | 72 | 83 | |
| % of fields of view with phenotypes | 12 | 35 | 100 | 100 | |
| Amy. fields of view with phenotypes | 2 | 1 | 17 | 17 | Amygdala |
| Total fields of view in Amy | 5 | 5 | 17 | 17 | |
| % of synapses with phenotypes | 7 | 3 | 44 | 81 | |
| % of fields of view with phenotypes | 40 | 20 | 100 | 100 | |
| Hyp. fields of view with phenotypes | 6 | 11 | 15 | 15 | Hypothalamus |
| Total fields of view in Hyp | 18 | 18 | 15 | 15 | |
| % of synapses with phenotypes | 20 | 27 | 67 | 77 | |
| % of fields of view with phenotypes | 33 | 61 | 100 | 100 | |

**Additional file 6:** NMDA receptor-dependent synaptic plasticity

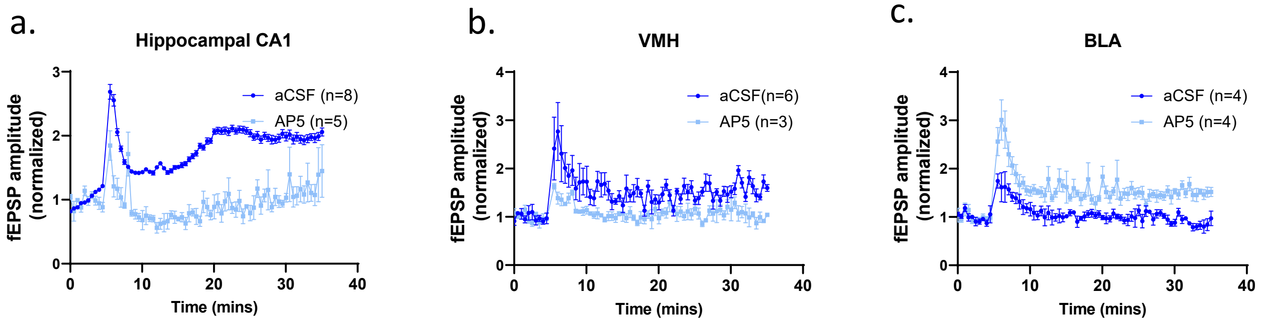


Tetani induced synaptic plasticity in the hippocampal CA1 (a), ventral media hypothalamus (VMH; b), and Basolateral amygdala (BLA; c) without or with AP5 blockage of NMDA receptors.

**Additional file 7:** NR1-containing NMDA receptor post-tetani

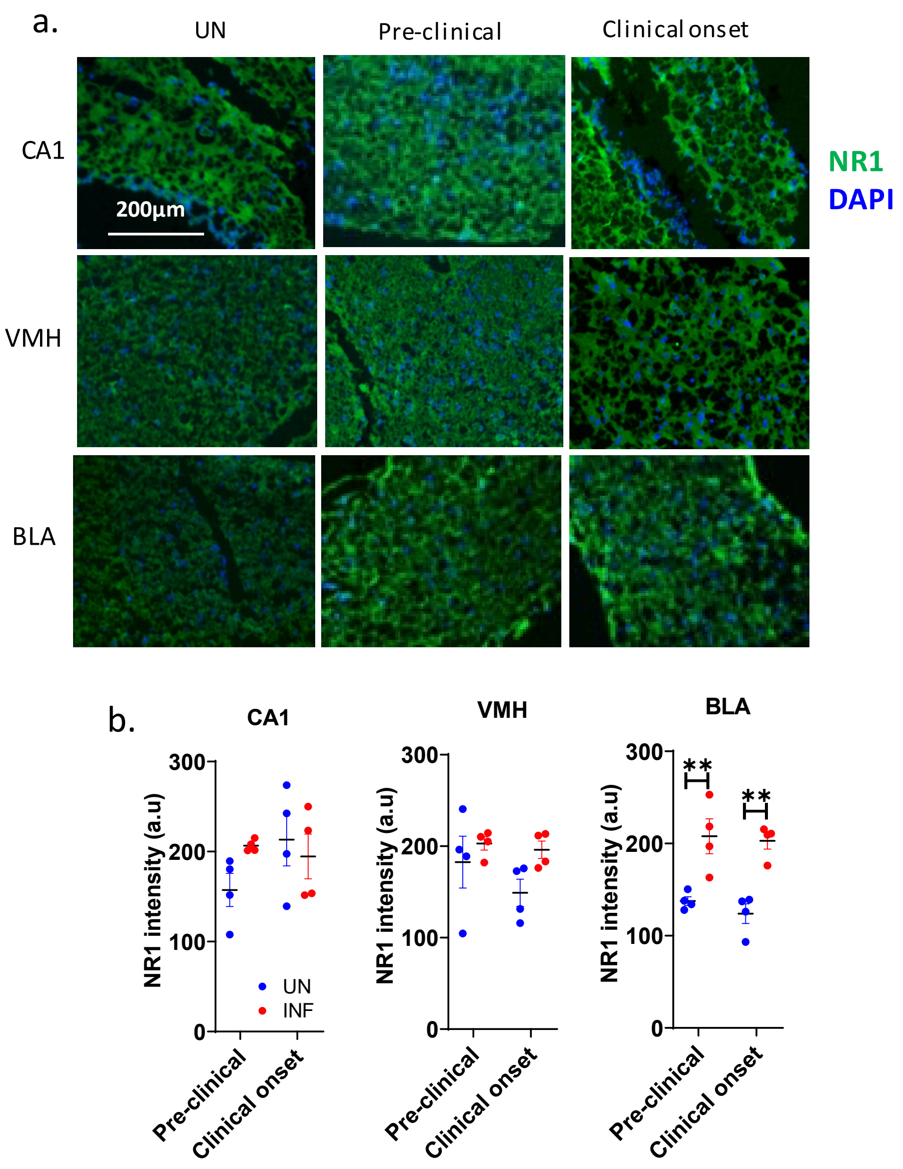


(a) NR1-containing NMDA receptors in the hippocampal CA1, VMH, and BLA, after synaptic plasticity induction (tetanic stimulations), in UN controls and INF mice at pre-clinical and clinical onset. (b) The quantifications of NR1 and each dot represents a mouse. Unpaired Student's t-test was used to compare the levels of NR1 between UN and INF samples. Data are presented as mean ± SEM. ** $p<0.01$.

**Additional file 8:** Changes in synaptic markers post-tetani

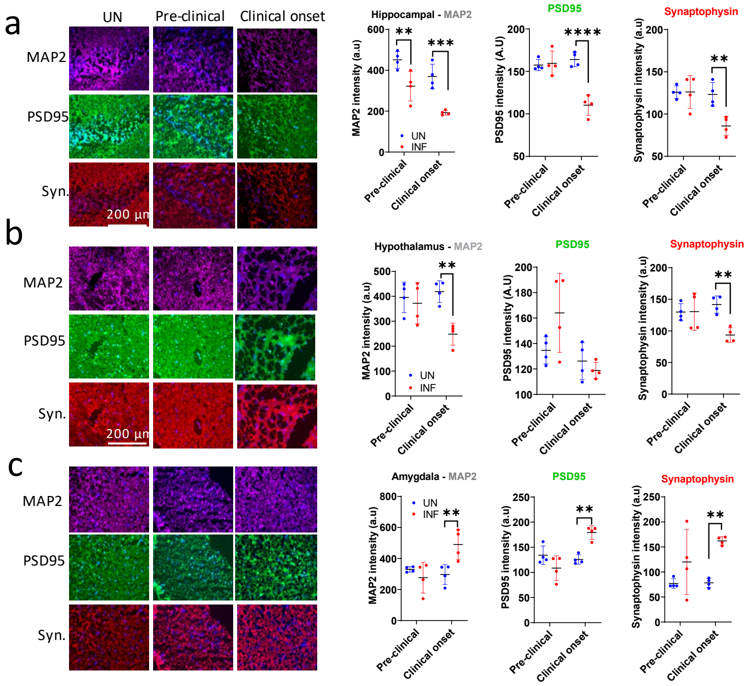


Immunofluorescence analysis of various synaptic markers, including MAP2 and PSD95 for post-synapses and synaptophysin for pre-synapses, in the 3 limbic regions (a – hippocampus ; b- hypothalamus; c-amygdala) following 30 minutes of the tetanic stimulations to induce long-term synaptic plasticity. The right panels show quantifications of the fluorescence intensity of the markers, comparing the infected samples from each disease timepoint to age-matched uninfected controls by an unpaired Student's t-test with Welch's correction. Data are presented as mean ± SEM. ** $p<0.01$, ***$p<0.001$, ****$p<0.0001$.

## **Additional file 9:** Pre-synapse size and count

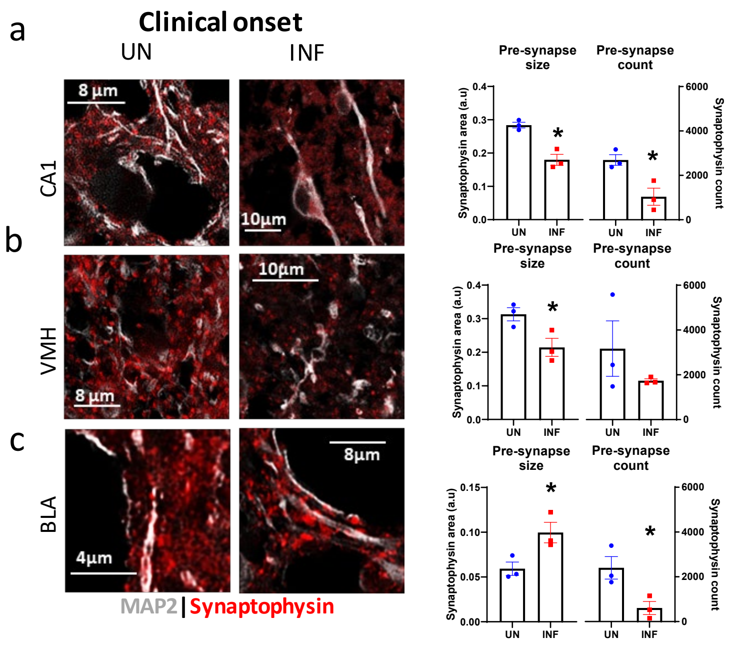


Estimating the size and quantity of pre-synapses in the three limbic regions from immunofluorescence images, as represented in left panels (a-hippocampal CA1; c-VMH; e-BLA), at clinical onset following the tetanic stimulations by measuring the area and number of synaptophysin (red). The right panels are quantifications comparing the synaptophysin area or count in infected tissues to that in age-matched uninfected controls by Student's t test with Welch's correction. Data are presented as mean ± SEM. *$p<0.05$

**Additional file 10:** GFAP level measuring astrogliosis

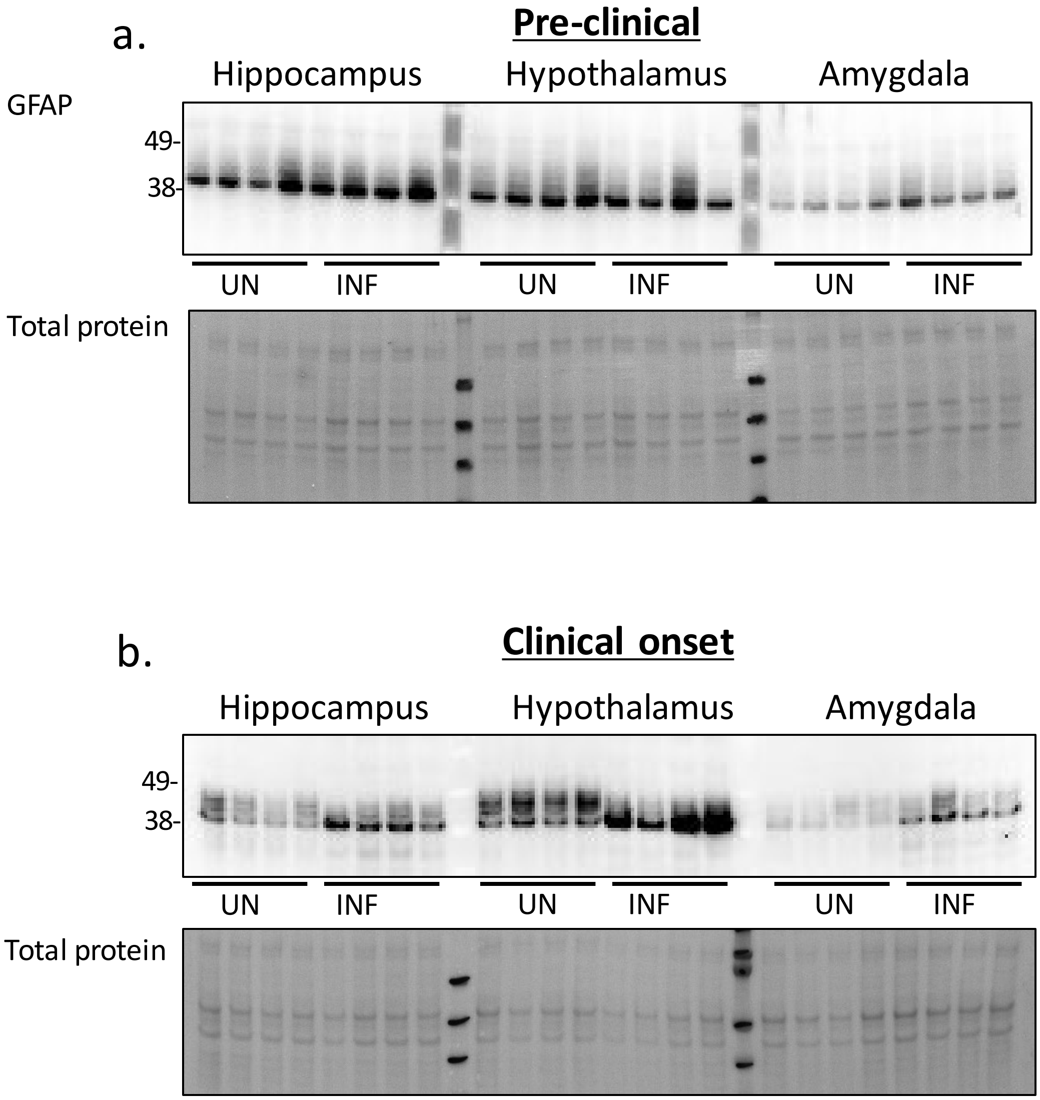


Western blotting analysis of astrogliosis marker GFAP in brain homogenates from 3 limbic regions at pre-clinical disease (a) and clinical onset (b) compared with age-matched uninfected controls. The bottom panels show the Coomassie stain for total protein and loading control.
