# Supplementary material for: Limbic system synaptic dysfunctions associated with prion disease onset
Source: Acta Neuropathol Commun. 2024 Dec 20;12:192. doi: 10.1186/s40478-024-01905-w (PMC11662616; doi:10.1186/s40478-024-01905-w)

Full blots

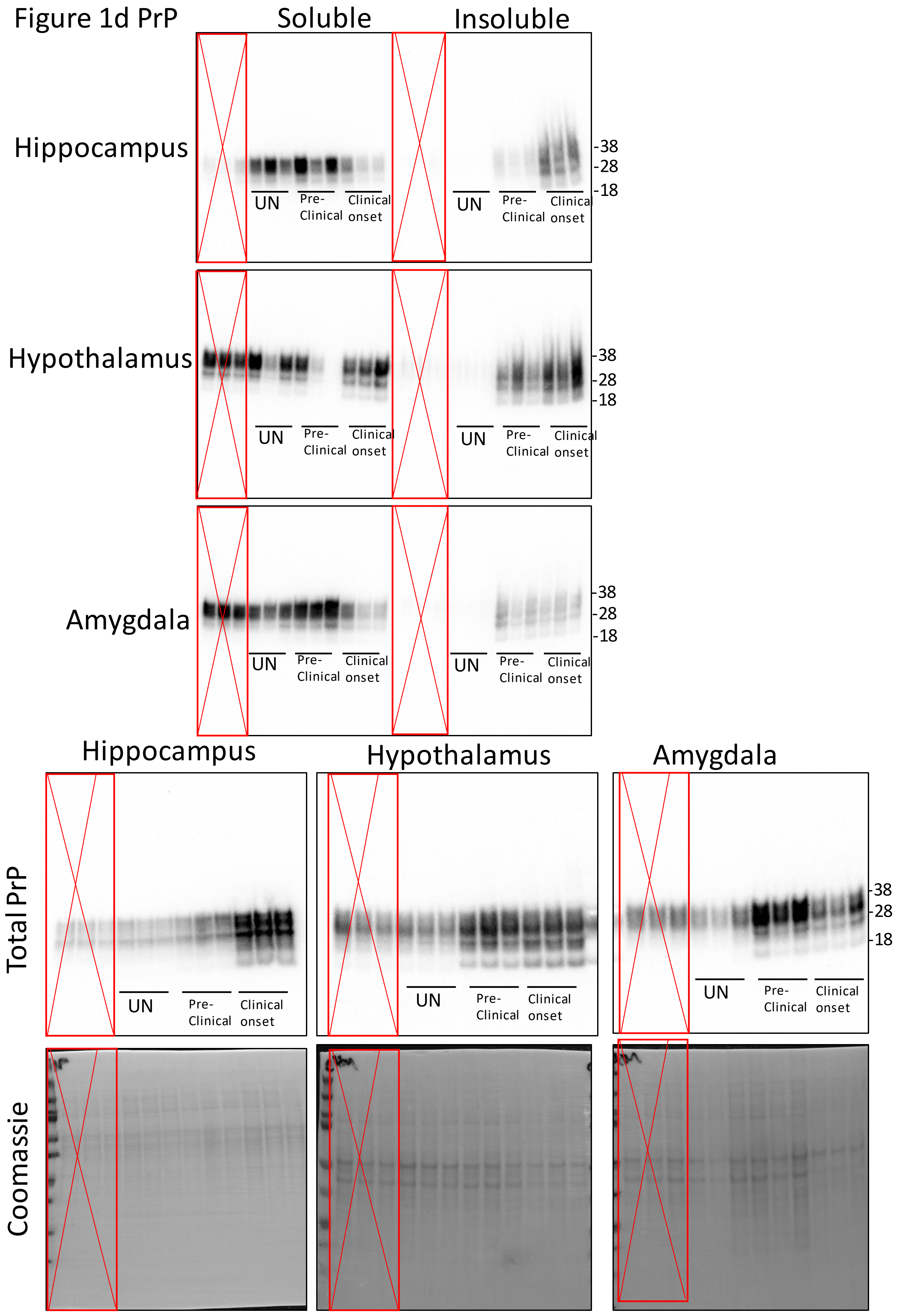

Figure 1d PrP

Figure 3 e Synaptophysin

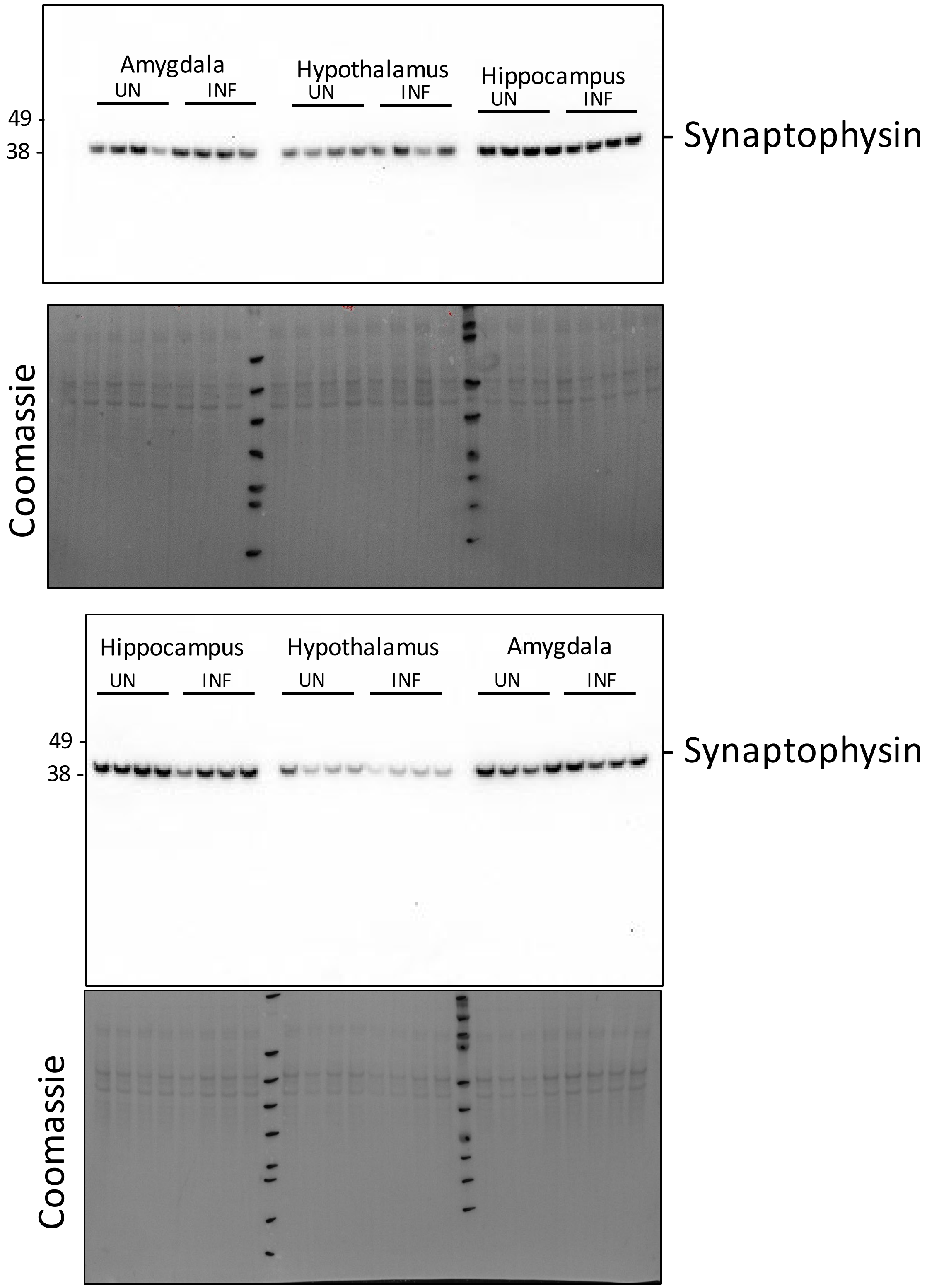

Figure 3 g PSD95

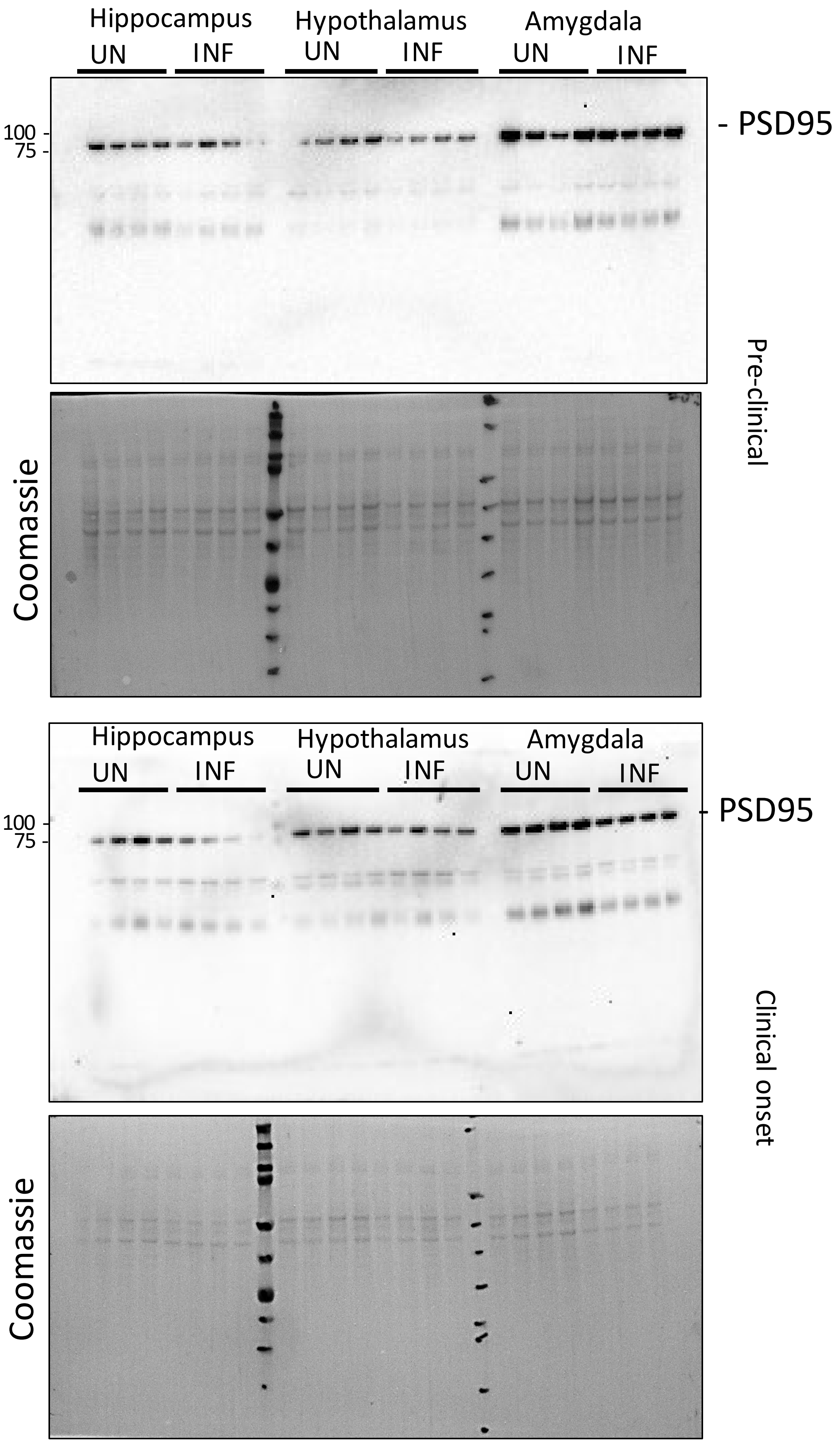

Figure 4 e NFL

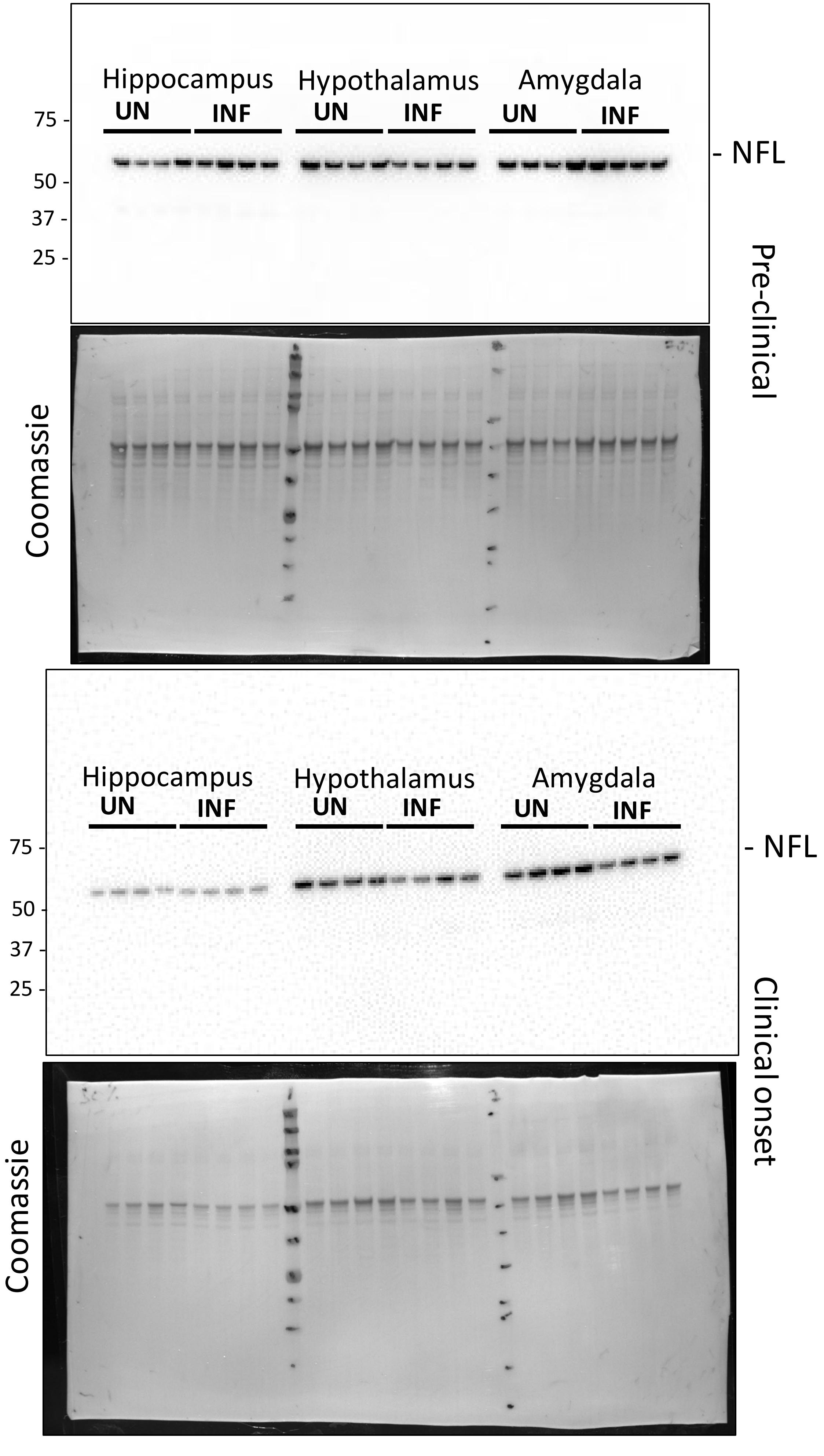

Figure 5: Western blotting for Synaptojanin 1

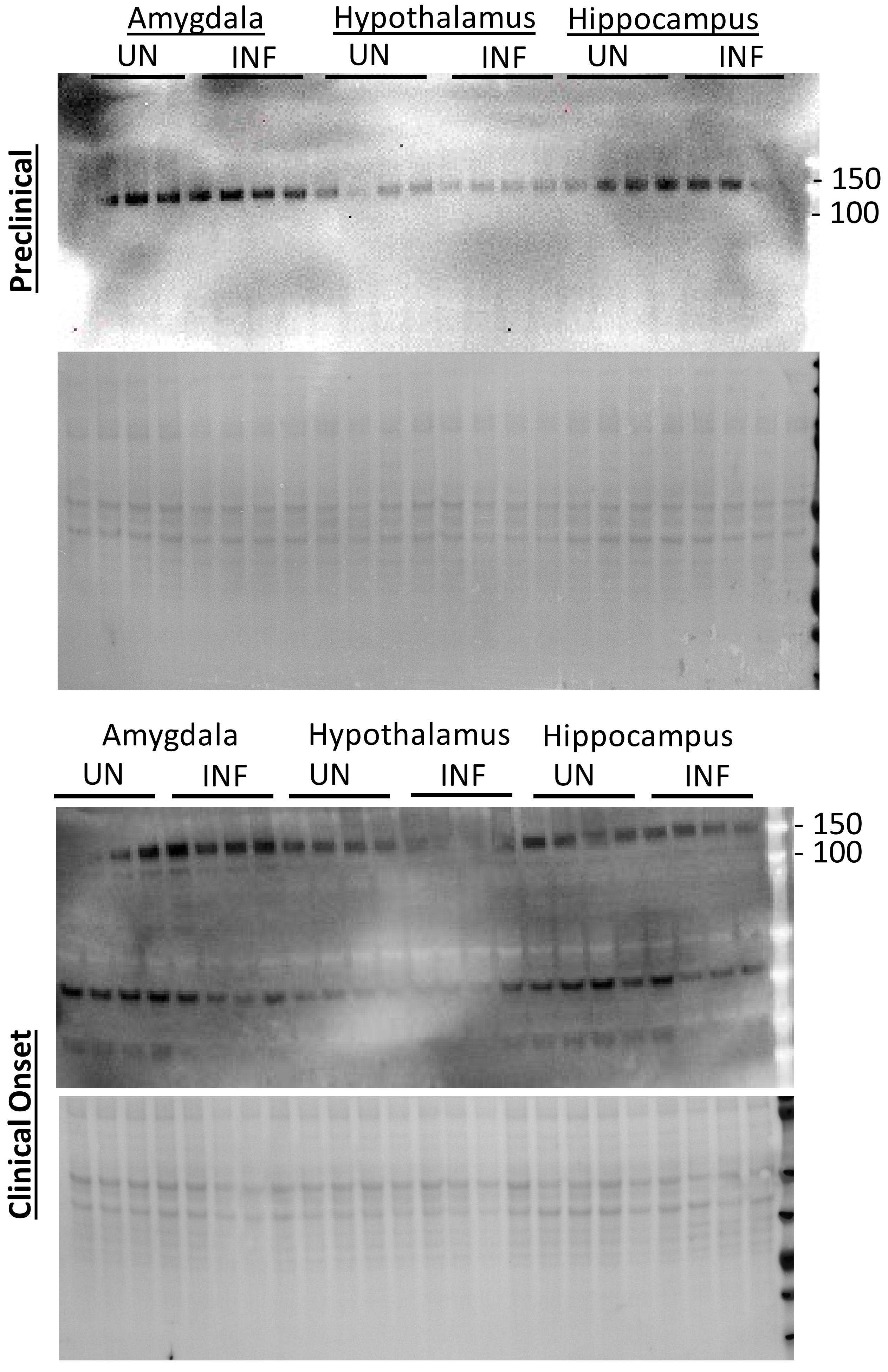

SI 2: Western blotting for NeuN

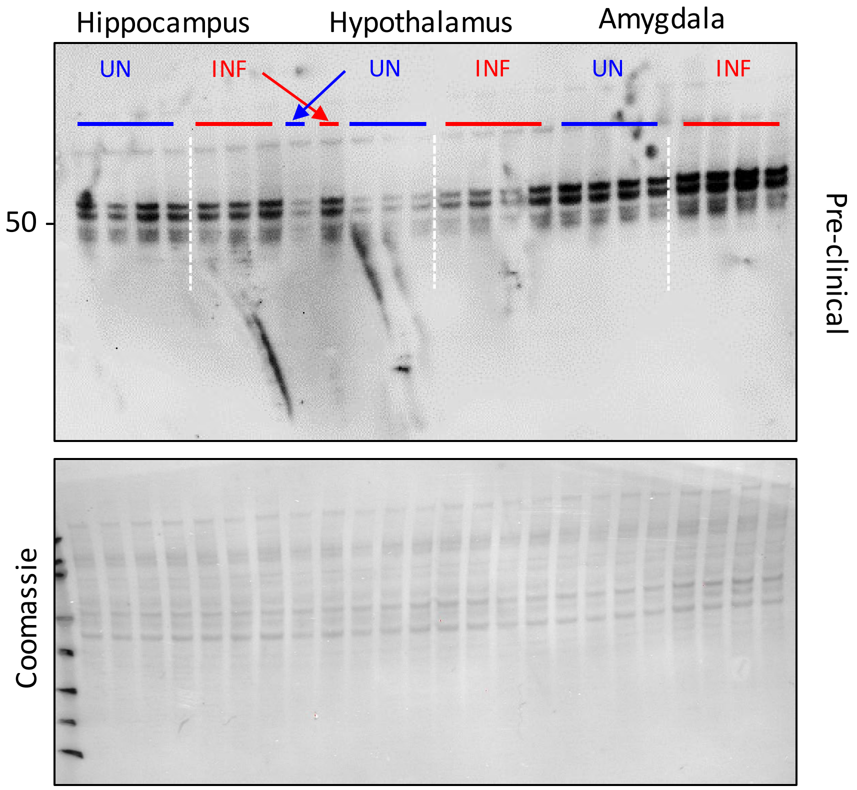

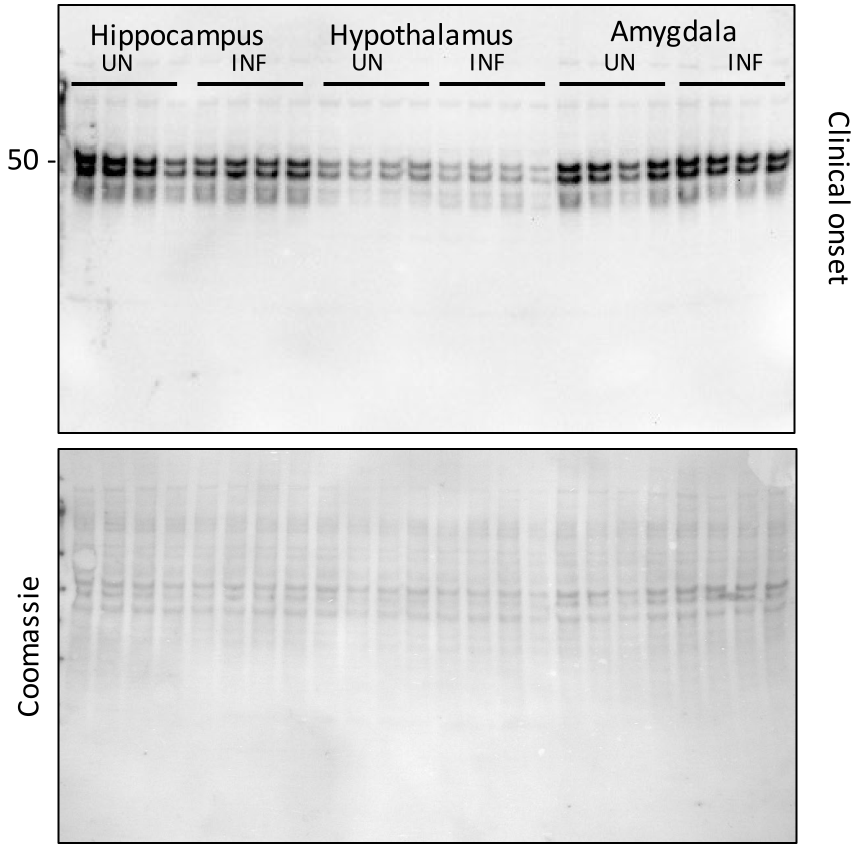

SI 8

GFAP level measuring astrogliosis

Pre-clinical

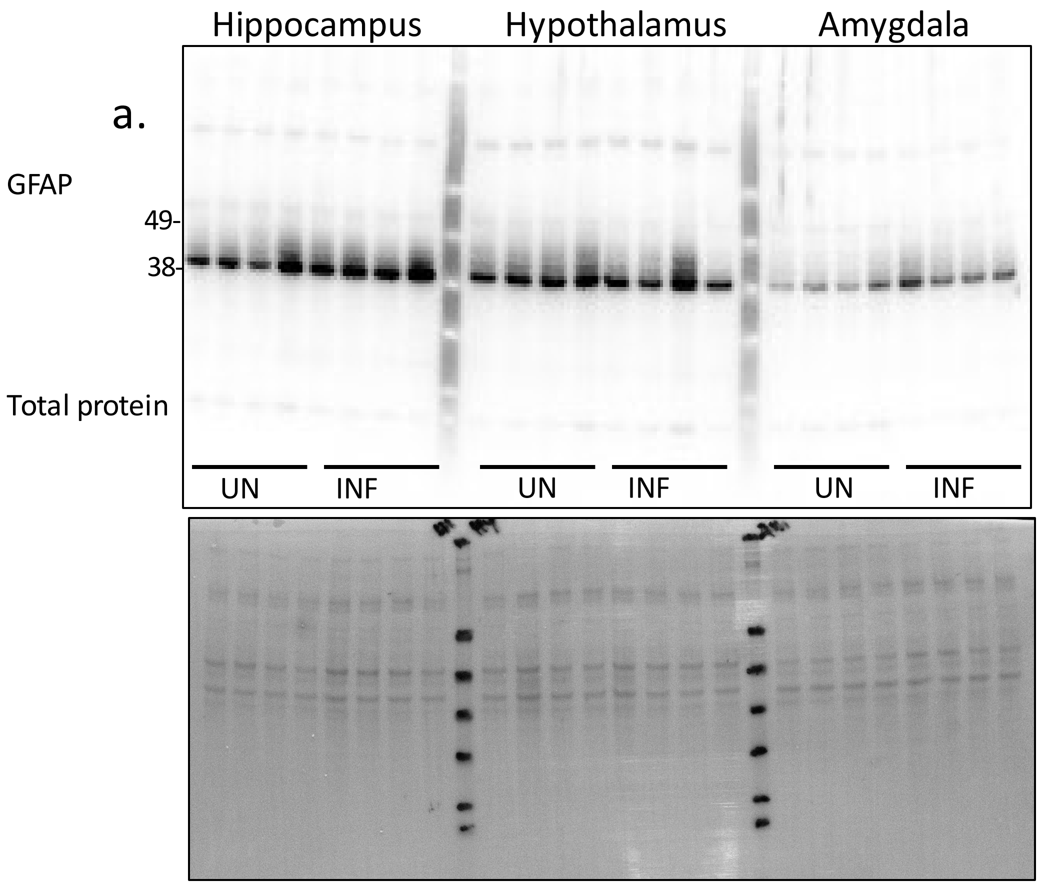

Clinical onset

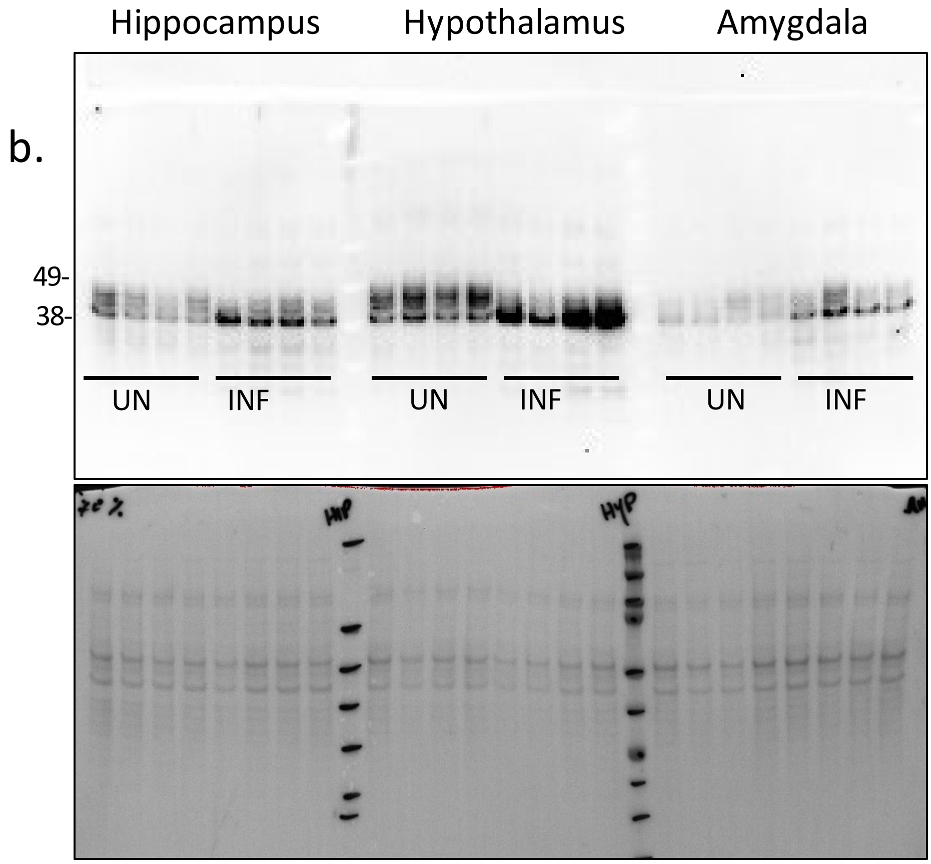

Supplement: Supplementary file 3 — Additional file 3. [file 40478_2024_1905_MOESM3_ESM.pdf]
