# Supplementary material for: Limbic system synaptic dysfunctions associated with prion disease onset
Source: Acta Neuropathol Commun. 2024 Dec 20;12:192. doi: 10.1186/s40478-024-01905-w (PMC11662616; doi:10.1186/s40478-024-01905-w)
Supplement: Supplementary file 4 — Additional file 4. [file 40478_2024_1905_MOESM4_ESM.pdf]

SI 1: Western blotting for NeuN

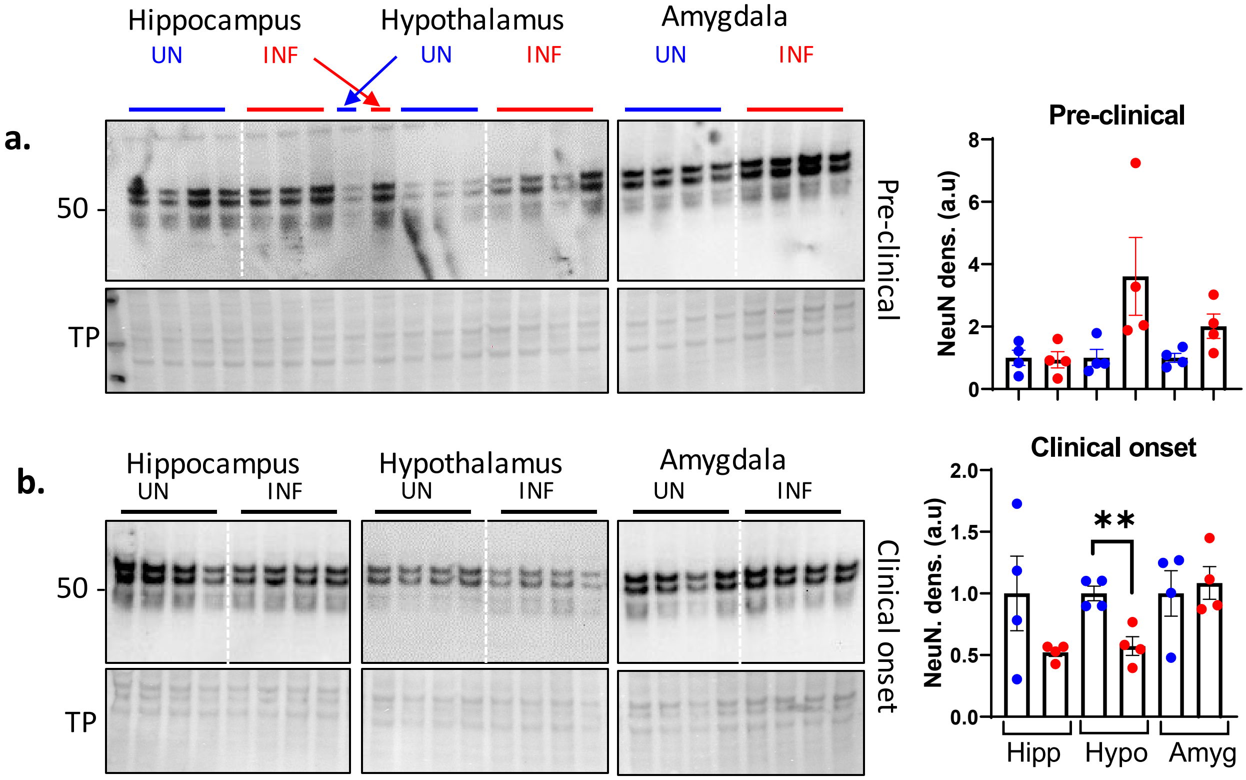

Western blotting analysis of NeuN level in the 3 limbic regions at pre-clinical stage(a) and clinical onset (b). Right panels are the quantifications comparing infected (INF) samples to age-matched uninfected (UN) controls by Unpaired Student's t test with Welch's correction. Data are presented as mean ± SEM. **$p<0.01$

SI 2: Burst representative traces

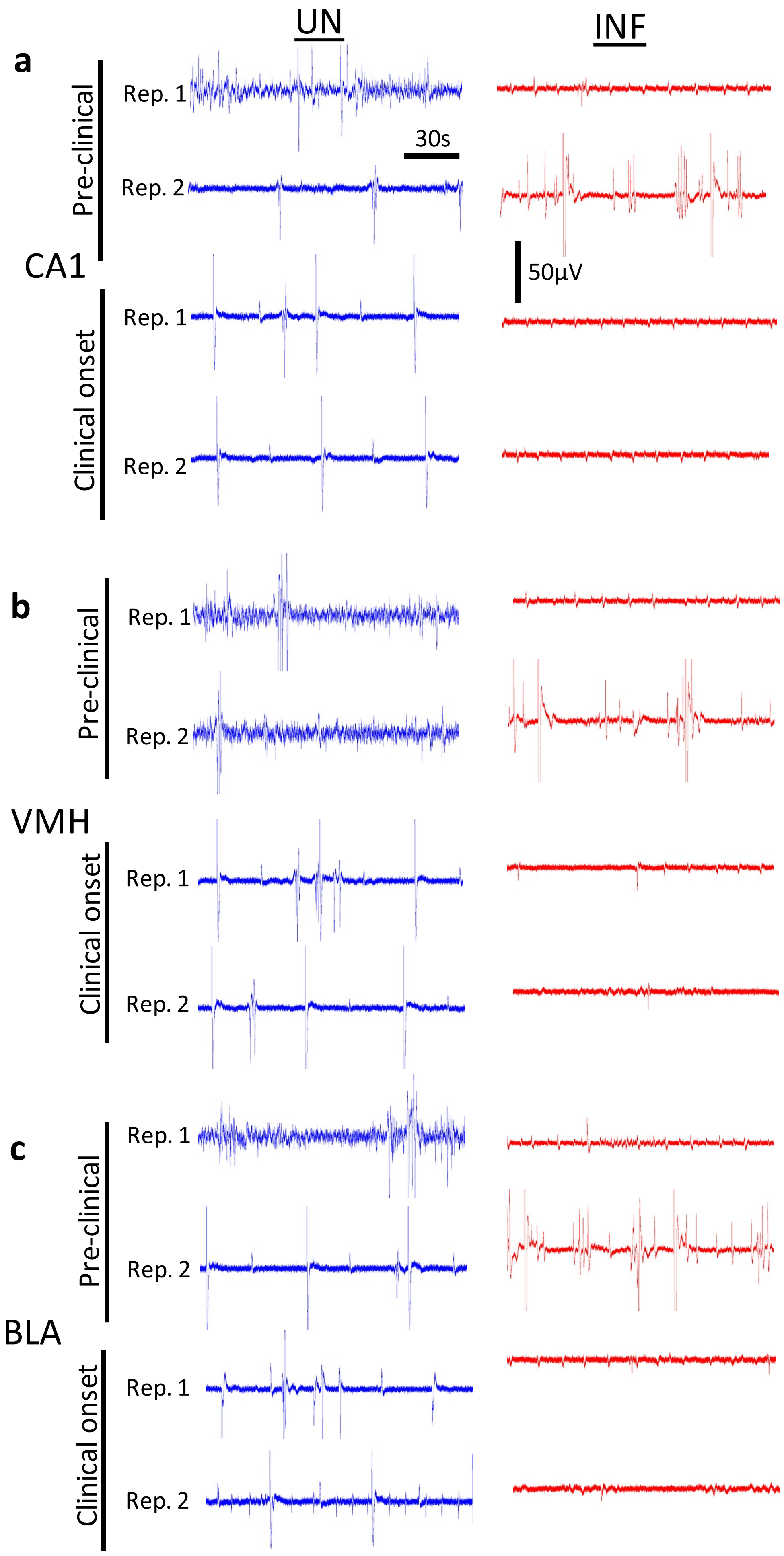

Representative (Rep.) traces of local field potentials with spikes and bursts in each limbic region (two Reps per group) at the pre-clinical stage and clinical onset compared with age-matched uninfected controls.

SI 3: Peak delta and gamma frequency

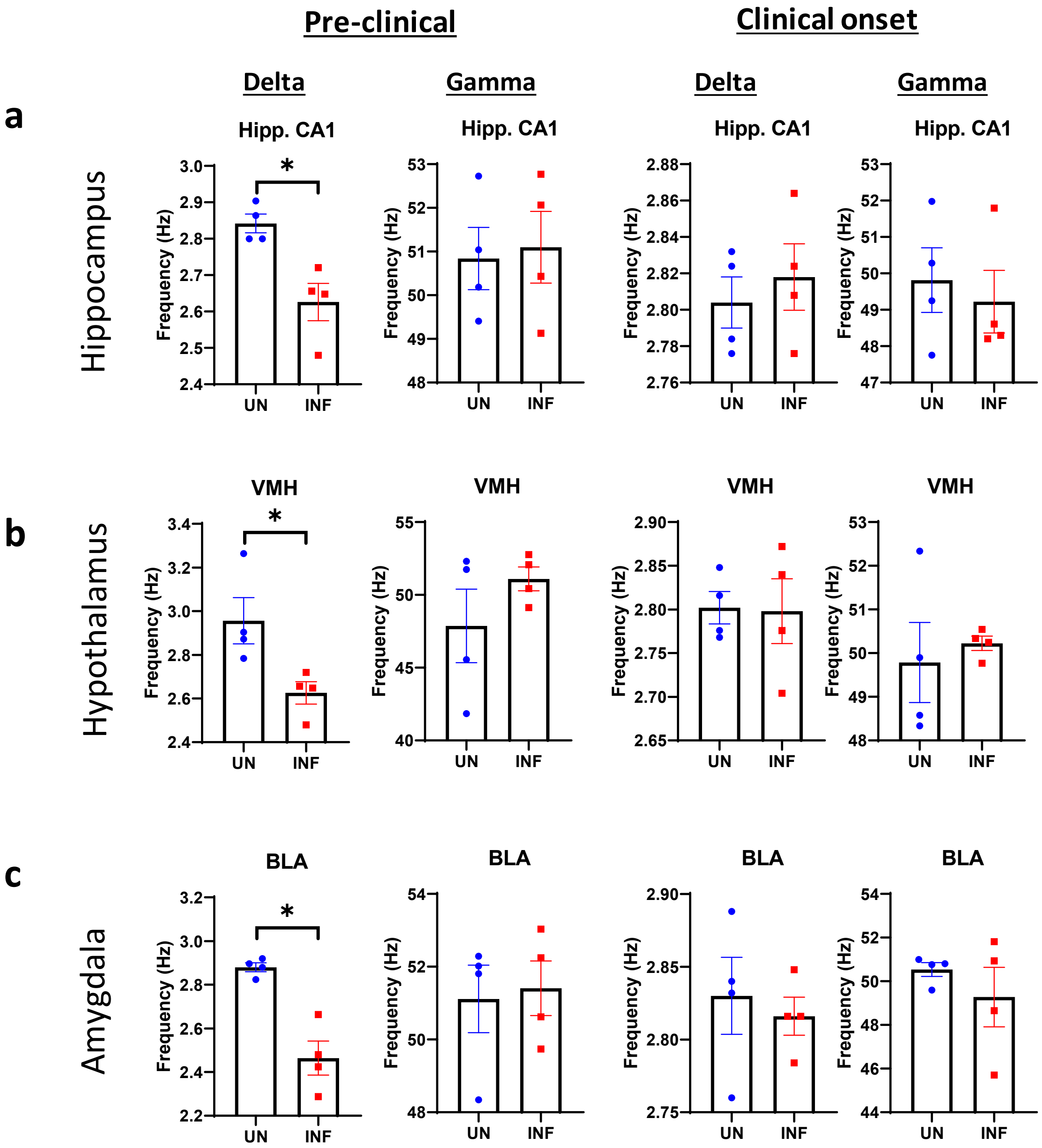

Peak frequency for delta and gamma oscillation in each limbic region at pre-clinical and clinical onset stages of disease compared with uninfected age-matched controls. Each dot represents a biological replicate, and data are presented as mean ±SEM. Mean peak frequency was compared between INF and UN by Student's t test with Welch's correction. ** p<0.05*

SI 4: Synaptojanin immunofluorescence analysis

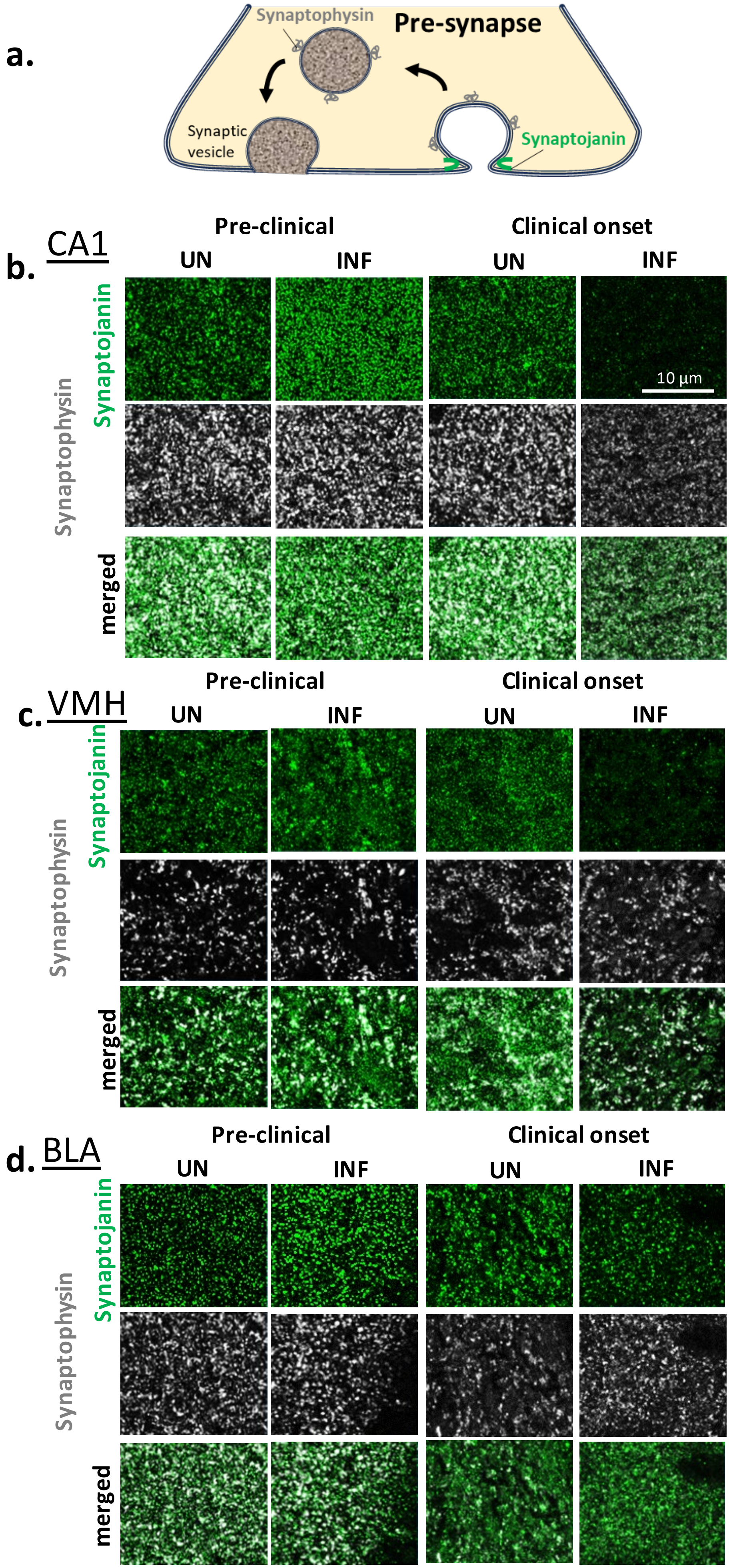

a. A schematic diagram of pre-synapse terminal with synaptophysin and synaptojanin localization.

b-d. Immunofluorescence analysis of synaptophysin and synaptojanin in the hippocampal CA1 (b), VMH (c), and BLA (d) at the pre-clinical stage and clinical onset relative to the age-matched uninfected controls.

SI 5 : NMDA receptor-dependent synaptic plasticity

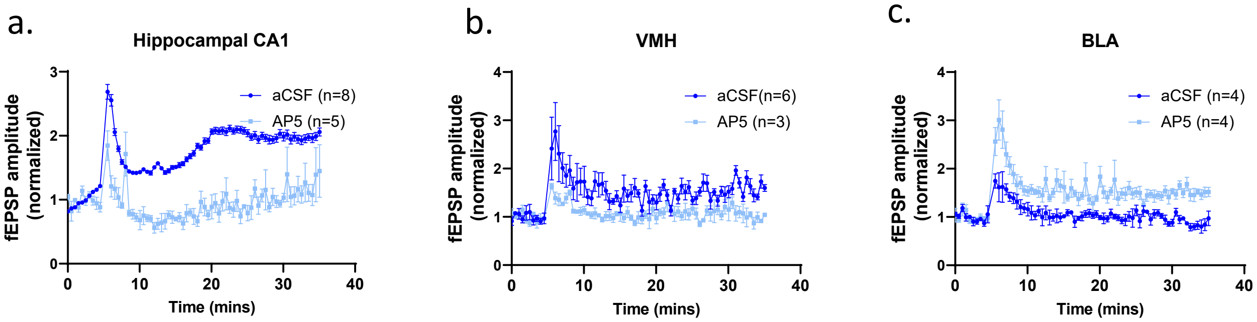

Tetani induced synaptic plasticity in the hippocampal CA1 (a), ventral media hypothalamus (VMH; b), and Basolateral amygdala (BLA; c) without or with AP5 blockage of NMDA receptors.

SI 6: NR1-containing NMDA receptor post-tetani

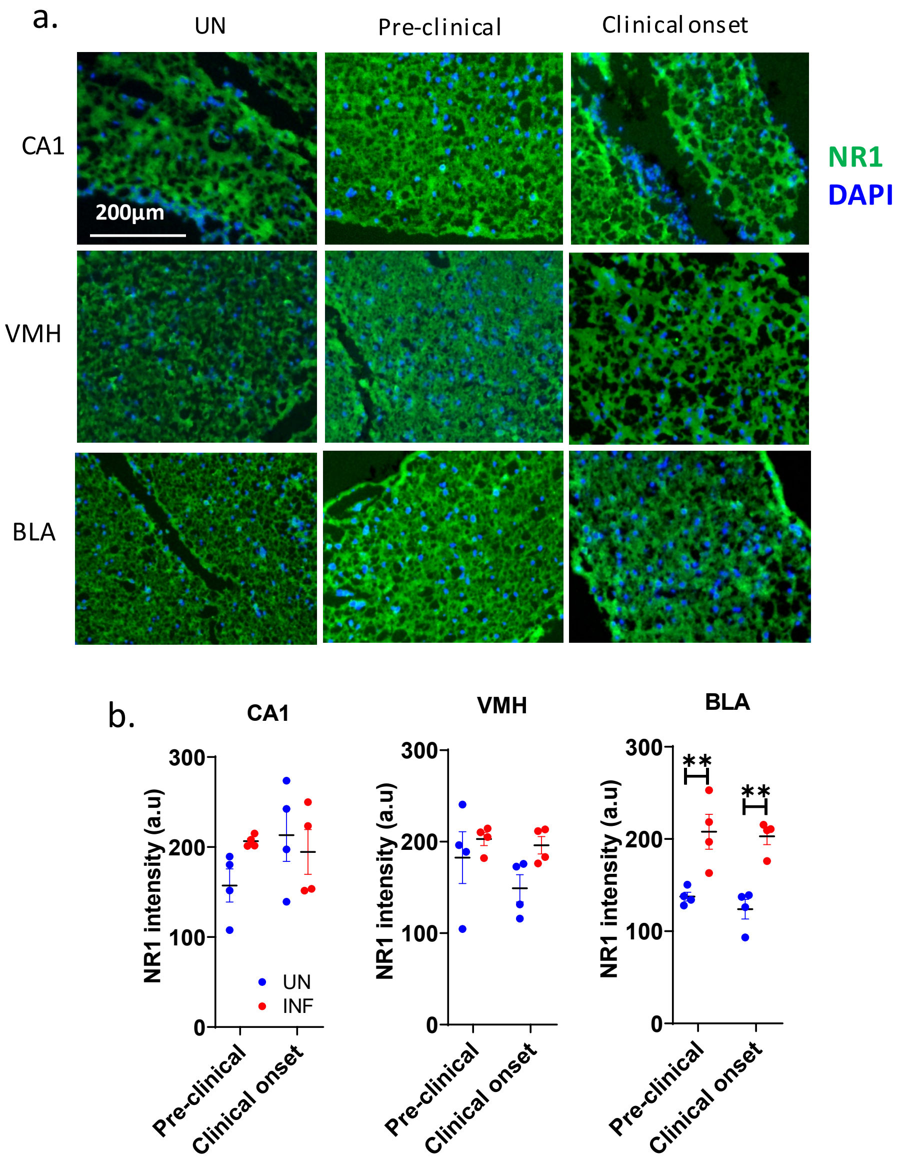

(a) NR1-containing NMDA receptors in the hippocampal CA1, VMH, and BLA, after synaptic plasticity induction (tetanic stimulations), in UN controls and INF mice at pre-clinical and clinical onset. (b) The quantifications of NR1 and each dot represents a mouse. Unpaired Student's t-test was used to compare the levels of NR1 between UN and INF samples. Data are presented as mean ± SEM. ** $p<0.01$.

SI 7: Changes in synaptic markers post-tetani

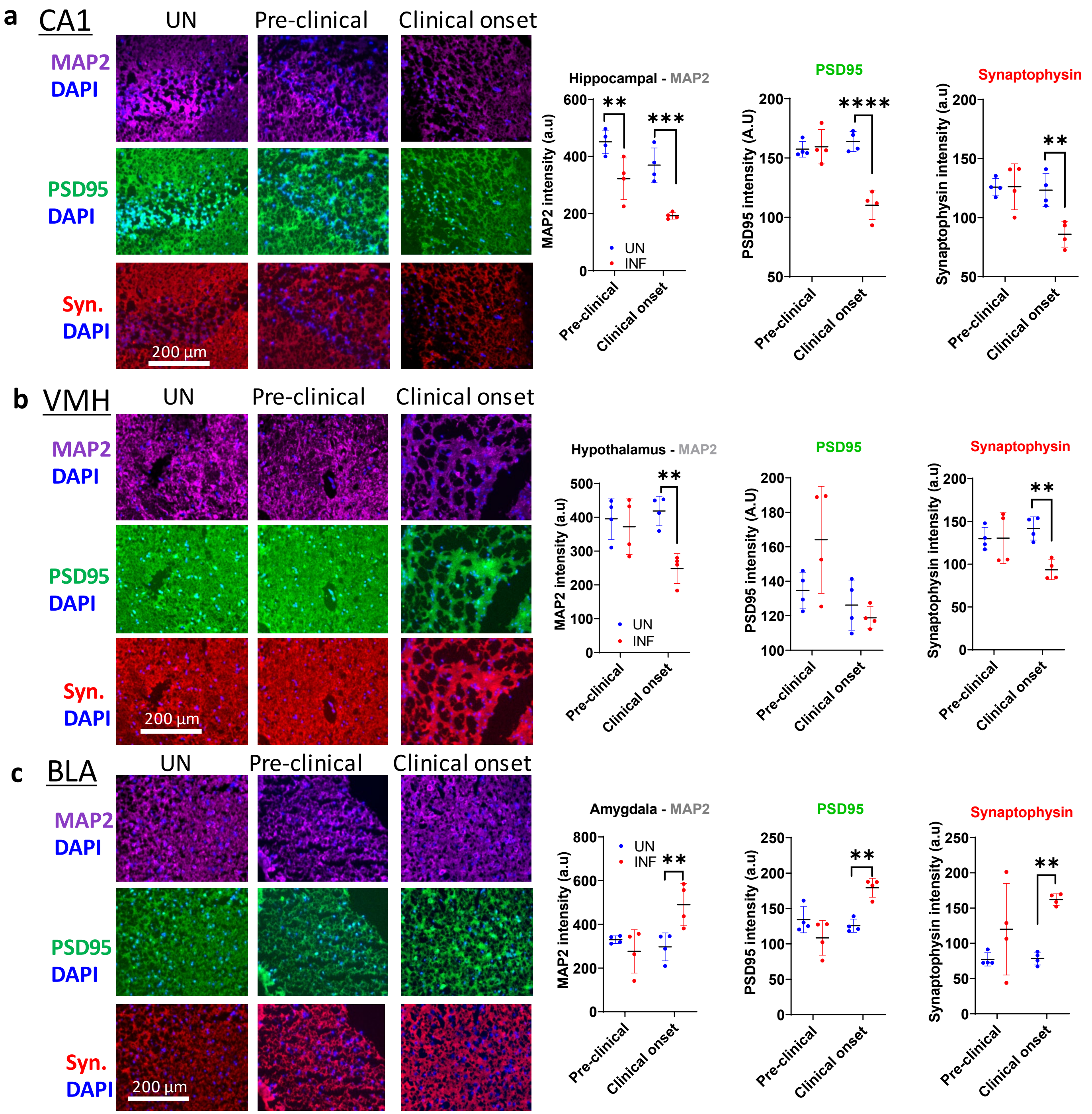

Immunofluorescence analysis of various synaptic markers, including MAP2 and PSD95 for post-synapses and synaptophysin for pre-synapses, in the 3 limbic regions (a – hippocampus ; b- hypothalamus; c-amygdala) following 30 minutes of the tetanic stimulations to induce long-term synaptic plasticity. The right panels show quantifications of the fluorescence intensity of the markers, comparing the infected samples from each disease timepoint to age-matched uninfected controls by an unpaired Student's t-test with Welch's correction. Data are presented as mean ± SEM. ** p<0.01, ***p<0.001, ****p<0.0001.

SI 8: Pre-synapse size and count

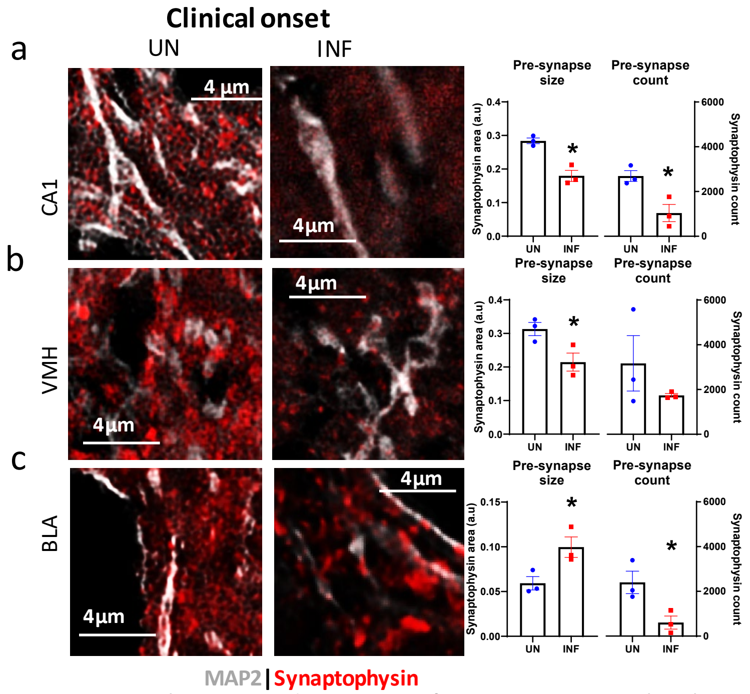

Estimating the size and quantity of pre-synapses in the three limbic regions from immunofluorescence images, as represented in left panels (a-hippocampal CA1; c-VMH; e-BLA), at clinical onset following the tetanic stimulations by measuring the area and number of synaptophysin (red). The right panels are quantifications comparing the synaptophysin area or count in infected tissues to that in age-matched uninfected controls by Student's t test with Welch's correction. Data are presented as mean ± SEM. $*p<0.05$

SI 9: GFAP level measuring astrogliosis

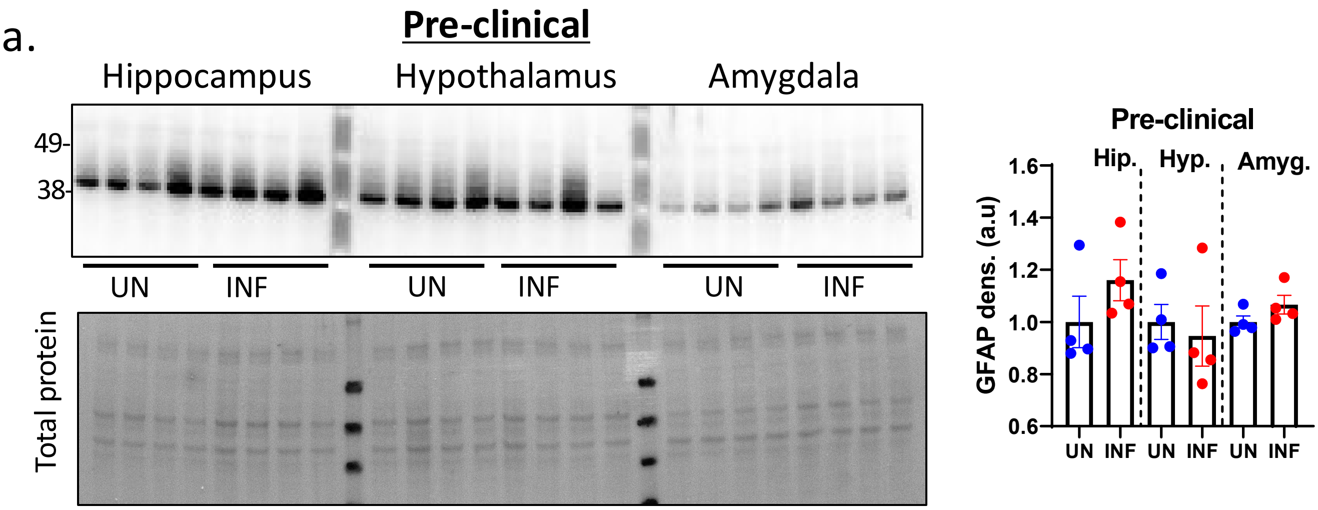

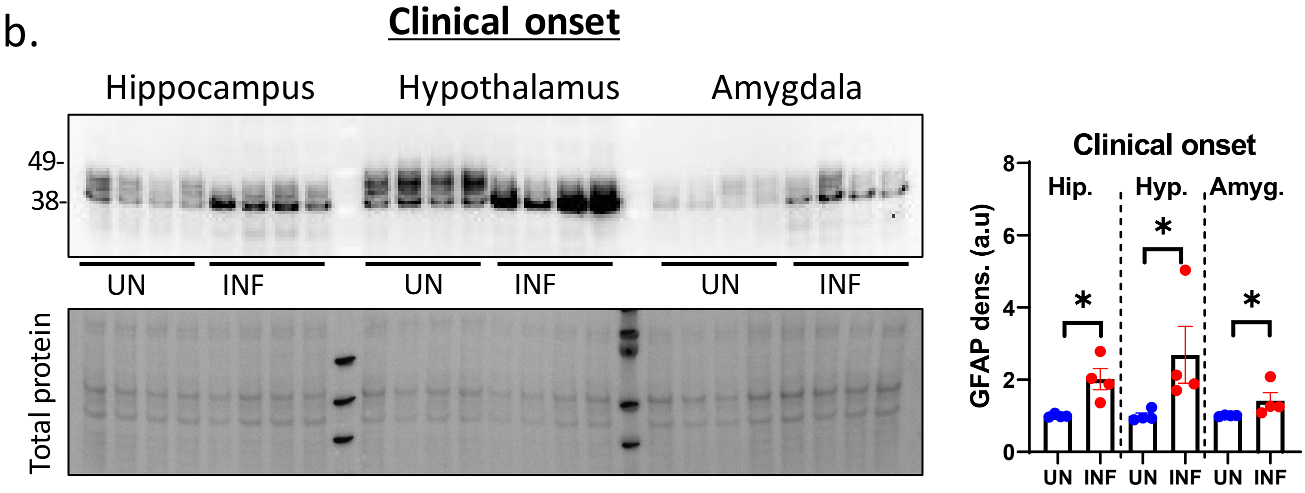

Western blotting analysis of astrogliosis marker GFAP in brain homogenates from 3 limbic regions at pre-clinical disease (a) and clinical onset (b) compared with age-matched uninfected controls. The bottom panels show the Coomassie stain for total protein and loading control. Left panels show quantification of GFAP in each time point (normalized to total protein). GFAP levels were compared between infected and uninfected samples within a region by Mann-Whitney test. Each dot represents a biological replicate. Data are presented as mean ± SEM. * *P<0.05*
